# Characterization and Expression Analysis of *ERF* Genes in *Fragaria vesca* Suggest Different Divergences of Tandem *ERF* Duplicates

**DOI:** 10.3389/fgene.2019.00805

**Published:** 2019-09-12

**Authors:** Xiaojing Wang, Shanshan Lin, Decai Liu, Quanzhi Wang, Richard McAvoy, Jing Ding, Yi Li

**Affiliations:** ^1^State Key Laboratory of Crop Genetics and Germplasm Enhancement and College of Horticulture, Nanjing Agricultural University, Nanjing, China; ^2^Engineering and Technology Center for Modern Horticulture, Jiangsu Vocational College of Agriculture and Forestry, Zhenjiang, China; ^3^Department of Plant Science and Landscape Architecture, University of Connecticut, Storrs, CT, United States

**Keywords:** *ERF* genes, tandem duplication, divergence, expression pattern, woodland strawberry

## Abstract

Ethylene-responsive factors (ERFs) play important roles in plant growth and development and in responses to abiotic stresses. However, little information was available about the *ERF* genes in woodland strawberry (*Fragaria vesca*), a genetic model plant for the *Fragaria* genus and Rosaceae family. In this study, 91 *FveERF* genes were identified, including 35 arrayed in tandem, indicating that tandem duplication is a major mechanism for the expansion of the FveERF family. According to their phylogenetic relationships with *AtERF*s from *Arabidopsis thaliana*, the tandem *FveERF* genes could be grouped into ancestral and lineage-specific tandem ones. The ancestral tandem *FveERF*s are likely derived from tandem duplications that occurred in the common ancestor of *F. vesca* and *A. thaliana*, whereas the lineage-specific ones are specifically present in the *F. vesca* lineage. The lineage-specific tandem *FveERF* duplicates are more conserved than the ancestral ones in sequence and structure. However, their expression in flowers and fruits is similarly diversified, indicating that tandem *FveERF*s have diverged rapidly after duplication in this respect. The lineage-specific tandem *FveERF*s display the same response patterns with only one exception under drought or cold, whereas the ancestral tandem ones are largely differentially expressed, suggesting that divergence of tandem *FveERF* expression under stress may have occurred later in the reproductive development. Our results provide evidence that the retention of tandem *FveERF* duplicates soon after their duplication may be related to their divergence in the regulation of reproductive development. In contrast, their further divergence in expression pattern likely contributes to plant response to abiotic stress.

## Introduction

Plants are sessile organisms and cannot escape from environmental stresses, which can negatively impact their survival, development, and productivity. As such, plants have evolved mechanisms to respond and adapt to stress at the physiological and biochemical levels (Figueiredo et al., 2012). Ethylene-responsive factors (ERFs) are transcription factors that have been shown to play critical roles in stress response and during plant growth and development (Brown et al., 2003; Chakravarthy et al., 2003; Agarwal et al., 2006; Chen G et al., 2008; Chen J. Q et al., 2008; Sun et al., 2014; Tan et al., 2018).

The ERF family belongs to the APETALA2/ERF (AP2/ERF) superfamily, which also contains the AP2 and RAV families (Weigel, 1995). ERF family proteins contain only one AP2/ERF domain, while the AP2 family contains proteins with a double tandem-repeated AP2 domain and the RAV family contains an additional B3 DNA-binding domain along with a single AP2/ERF domain (Matías-Hernández et al., 2014). In *Arabidopsis thaliana*, the ERF family is divided into 10 groups (I to X) based on phylogeny and gene/protein structure analyses (Nakano et al., 2006). ERF family genes have diverse expression patterns during plant growth and development (Wilson et al., 1996; Liu et al., 1998; Banno et al., 2001), as well as in response to abiotic stresses, such as drought, cold, and high salinity (Song et al., 2005; Novillo et al., 2007; Golldack et al., 2011; Licausi et al., 2013).

Tandem gene duplication is one of the main gene-duplication mechanisms in eukaryotes and has contributed to the prevalence of gene family clusters (Fortna et al., 2004; Fan et al., 2008). The number of tandem duplicates in plants varies from 451 (4.6% of gene content) in *Craspedia variabilis* to 16,602 (26.1% of gene content) in apple (*Malus* × *domestica*) (Yu et al., 2015). Genome-wide analysis in *A. thaliana* has revealed that genes that expanded mainly through tandem duplication tend to be involved in plant responses to abiotic and biotic stresses (Hanada et al., 2008). To the contrary, transcription factors including *ERF*s are preferentially retained after whole-genome duplication (WGD) rather than tandem duplication (Maere et al., 2005; Jourda et al., 2014; Charfeddine et al., 2015). Nevertheless, studies in *A. thaliana* and cucumber show that tandem-duplication events have also played an important role in the expansion of the *ERF* gene family (Nakano et al., 2006; Hu and Liu, 2011).

Duplicate genes experience relaxed negative selection following duplication (Carretero-Paulet and Fares 2012). Increased rates of evolution, *via* divergence of gene sequence, structure, and so forth, have been observed in duplicate gene copies (Carretero-Paulet and Fares, 2012; Wang et al., 2013). Divergence in expression patterns of duplicate genes is affected by their functional categories, duplication mechanisms, species, and other factors (Wang et al., 2012a). Studies in *A. thaliana* and rice show that expression divergence among tandem duplicates occurs shortly after duplication (Ganko et al., 2007; Li et al., 2009), and its overall level is similar to that of WGD duplicates but lower than that of duplicates from other mechanisms (Wang et al., 2012b). There is no significant correlation between expression divergence of tandem duplicates and their synonymous substitution rates, a proxy for the time of duplication (Ganko et al., 2007; Panchy et al., 2016). This indicates that young and old tandem duplicates have a similar level of expression divergence. However, this observation is mainly based on expression analysis in developmental tissues/organs; whether it is the case for expression patterns under stressed conditions remains unclear.

Cultivated strawberry (*Fragaria* × *ananassa*) is a popular crop worldwide; however, genetic analysis of cultivated strawberry is extremely complicated due to its octoploid genome (2*n* = 8*x* = 56), with as many as four diploid ancestors. Nowadays, woodland strawberry (*Fragaria vesca*) is emerging as a model fruit crop plant species. It has a small diploid genome (240 Mb, 2*n* = 2*x* = 14) with a widely available genome sequence (Shulaev et al., 2011) and a short reproductive cycle (14–15 weeks in climate-controlled greenhouses). In this study, we performed a comprehensive analysis of the ERF family in *F. vesca*, including phylogeny, chromosomal localization, gene structure, motif, duplication mechanism, and expression profiling. Tandem *FveERF* genes were grouped into ancestral and lineage-specific tandem ones and subjected to expression pattern analysis during reproductive development and in response to drought or cold stress. The results of this study should be useful towards future analyses of the divergence and functions of *ERF* genes, particularly tandem duplicated *ERF* genes in strawberry.

## Materials and Methods

### Identification of *AP2/ERF* Genes in *F. vesca*

The *F. vesca* genome sequence and corresponding annotations were downloaded from the DOE Joint Genome Institute website (http://genome.jgi.doe.gov/). First, the full alignment file for the AP2 domain (PF00847) obtained from the Pfam database (Finn et al., 2016) was used to build an HMM file using the HMMER3 software package (Eddy, 1998). Second, HMM searches were performed against the local protein databases of *F. vesca* using the HMMER3 package. Moreover, we checked the physical localizations of all candidate genes and rejected redundant sequences with the same chromosome location and short proteins (length < 100 aa). Finally, sequences of all matching proteins were again analyzed in the Pfam database to verify the presence of AP2 domains. AP2 domains were also detected by the SMART (http://smart.embl-heidelberg.de/) database with an *E*-value cutoff of 10^−10^. After the above four steps, the identified protein sequences that contained the core domains (AP2 domain) of known AP2/ERFs were regarded as putative homologs in the study.

### Gene Structure and Chromosomal Localization of *FveERF* Genes

Exon/intron information and chromosomal location of *FveERF* genes were extracted from the *F. vesca* genome annotation database. The data were then plotted using the MapInspect software (http://mapinspect.software.informer.com/). Tandem duplicate *FveERF*s were defined as *FveERF*s in any gene pair that is located within 100 kb of each other and separated by no more than 10 non-homologous intervening genes (Hanada et al., 2008). Fgenesh (http://www.softberry.com) was used to re-annotate the intergenic regions between putative tandem *FveERF* duplicates, to clarify whether there are any unannotated intervening genes. If the number of non-homologous intervening genes based on genome annotation and our re-annotation results is no more than 10, we consider the pair of *FveERF*s as tandem duplicate genes. The tandem *ERF* genes in *Malus* × *domestica*, *Prunus mume*, *Populus trichocarpa*, *Brassica rapa*, *Vitis vinifera*, *Solanum tuberosum*, and *Oryza sativa* were identified based on the same criterion without re-annotation of the intergenic regions. Besides, the tandem *AtERF* genes in *A. thaliana* were retrieved from the study by Nakano et al. (2006).

### Phylogenetic Analyses of *ERF* Genes from *F. vesca* and *A. thaliana*

The sequences of 146 AP2/ERF proteins from *A. thaliana*, identified by Nakano et al. (2006), were used for comparative analysis in the study. Full-length amino acid sequences of the AP2/ERFs from *F. vesca* and *A. thaliana* were aligned using ClustalX2.0 (Larkin et al., 2007) and MAFFT [version 7, Katoh and Standley (2013)], respectively, with default parameters. A maximum-likelihood (ML) phylogeny based on the ClustalX alignment (**Figure S1A**) and a aBayes phylogeny based on the MAFFT alignment (**Figure S1B**) were constructed, respectively, using the PhyML software (version 3.0, Guindon et al., 2010). Both phylogenies show a same grouping of the FveAP2/ERF superfamily. Next, full-length amino acid sequences of the identified FveERFs were aligned with those of the AtERFs using ClustalX2.0 and MAFFT, respectively. The JTT+G+I substitution model was identified as the optimal model of amino acid sequence evolution using the program MODELGENERATOR (Keane et al., 2006) with four gamma categories (Jones et al., 1992). ML phylogenies based on the ClustalX (**Figure 2**) and MAFFT alignments and an aBayes phylogeny based on the MAFFT alignment (**Figure S2**) were constructed, respectively, using the PhyML software with the model. The reliabilities of the ML phylogenies and the aBayes phylogenies were tested using bootstrapping with 100 replicates and Bayes posterior probabilities, respectively.

### Motif Analysis of FveERF Proteins

The MEME5.0.1 online program (http://meme-suite.org/) was used for the identification of motifs in the FveERF protein sequences. The optimized parameters were employed for the analysis as follows: number of repetitions: any; maximum number of motifs: 15; and the optimum width of each motif: between 6 and 50 residues (Bailey et al., 2015).

### Synteny Analysis

Synteny analysis of the *F. vesca* genome was conducted locally using a method similar to the one used by the plant genome duplication database (PGDD, http://chibba.agtec.uga.edu/duplication/, Lee et al., 2013). First, BLASTP was performed to search for potential homologous gene pairs (*E* < 10^−5^, top five matches) in *F. vesca* genome. Then, the homologous pairs were used as input for MCScanX to identify syntenic chains and types of duplication mechanisms (Tang et al., 2008; Wang et al., 2012a).

### Calculation of Pi, Ka, Ks, and Ka/Ks Values of *FveERF* Genes

Pairwise nucleotide divergence among paralogs was estimated by Pi using DnaSP v4.0 (Rozas et al. 2003). To analyze evolutionary rates of tandem duplicate *FveERF*s, the coding sequences of *FveERF* genes were aligned on the basis of the corresponding aligned protein sequences using the PAL2NAL software (Suyama et al. 2006). The ratio of nonsynonymous substitutions per nonsynonymous site (Ka) to synonymous substitutions per synonymous site (Ks) in tandem gene pairs was calculated by using the yn00 program of the PAML package (Yang, 1997). Generally, a Ka/Ks ratio >1 indicates positive selection, and a ratio <1 indicates negative or purifying selection, while a ratio of 1 indicates neutral evolution.

### Expression Pattern of FveERF Family Genes and Correlation Analysis

Expression data of *FveERF* genes among different stages and tissues of *F. vesca* flowers and early fruits were retrieved from the SGR database (http://bioinformatics.towson.edu/strawberry/). The heat map was created using the log2 “relative RPKM (reads per kilobase per million) values” of individual *FveERF* genes. For a detailed description of the stages and tissues, please see http://bioinformatics.towson.edu/strawberry/newpage/Tissue_Description.aspx. According to Kang et al. (2013), a gene with an RPKM value lower than 0.3 was regarded not to be expressed in a certain stage or tissue. A gene with RPKM values higher than 0.3 in at least two stages or tissues was regarded as an expressed gene during flower or early-fruit development. Statistical tests of differences between expression levels of tandem/clustered and other *FveERF*s, and of ancestral and lineage-specific tandem *FveERF*s were performed using *t*-test. The correlation between expression patterns of tandem duplicate genes was evaluated by calculating correlation coefficients of the expression data, where the RPKM values lower than 0.3 was not included.

### Growth Conditions, Plant Material Collection, and Abiotic Treatments

All plant material was collected from a seventh-generation inbred line of *F. vesca* ‘Ruegen’ (kindly provided by Janet Slovin). Plants were grown in 10 cm × 10 cm pots in a growth chamber on a 16-h light (22 °C)/8-h dark (20 °C) cycle with 65% relative humidity. Light (∼160 µmol m^−2^ s^−1^) was supplied by sodium lamps. Four developmental stages of Ruegen receptacles were collected for quantitative PCR (qPCR) analysis: little white (white flesh with green achenes, ∼20 DPA), pre-turning (white flesh with red achenes, ∼DPA), pink (light pink flesh with red achenes, ∼27 DPA), and red (flesh is all red, ∼29 DPA) stages. All samples were collected and immediately put into liquid nitrogen.

Prior to abiotic stress treatments, strawberry seedlings were grown on solid MS media in the growth chamber on a 16-h light (22 °C)/8-h dark (20 °C) cycle for 1 month. Cold stress treatments were carried out as described in Gu et al. (2016). For drought stress treatments, the seedlings were removed from the media, placed on filter paper under dim light and 30% humidity, and collected after 1, 3, and 8 h of dehydration. Following abiotic stress treatment, plant materials were immediately put into liquid nitrogen prior to RNA processing.

### RNA Extraction and Quantitative RT-PCR (qRT-PCR) Analysis

The RNA of stress-treated seedlings was isolated using a TaKaRa MiniBEST Plant RNA Extraction Kit. Nine *FveERF*s from all the lineage-specific tandem repeats (all six genes from two tandem repeats plus three genes randomly selected from the six-gene tandem repeat, mrna08071–mrna08075 and mrna08077, **Table S1**) were selected for qRT-PCR analyses. As most lineage-specific tandem *FveERF*s belong to group 9, the nine ancestral tandem *FveERF*s in groups 9 and 10 were selected for comparison. qRT-PCR primers for these genes are listed in **Table S1**. Expression of the four lineage-specific tandem *FveERF*s that are very lowly expressed in early fruits (mrna04911, mrna04913, mrna08873, and mrna08876) was not examined in the fruit-ripening stages. qRT-PCR was performed using SYBR Premix Ex Tag (TaKaRa) using cDNA as the template. Results were analyzed using the ^−ΔΔ^CT method with *GAPDH* gene expression as an internal reference (Livak and Schmittgen, 2001; Amil-Ruiz et al., 2013). Three biological and three technical replicates were used.

## Results

### Genome-Wide Identification of *ERF* Genes in *F. vesca*

To identify the ERF family members in *F. vesca*, the full-length alignment of the AP2/ERF domain (PF00847) was downloaded and used to search the *F. vesca* proteome. A total of 115 proteins were considered as AP2/ERF candidates, containing at least one AP2/ERF domain. Maximum-likelihood (ML, **Figure S1A**) and aBayes (**Figure S1B**) phylogenetic trees were created, respectively, based on the ClustalX and MAFFT alignments of these 115 AP2/ERF candidates and 146 AP2/ERF proteins from *A. thaliana*. Both phylogenies show the same grouping of the AP2/ERF superfamily in *F. vesca*. According to these phylogenies, as well as their domain compositions, 91 proteins were classified as *F. vesca* ERFs (FveERFs), and the other 24 proteins were grouped to the AP2, RAV families or soloists (**Table S2**).

**Figure 1:**
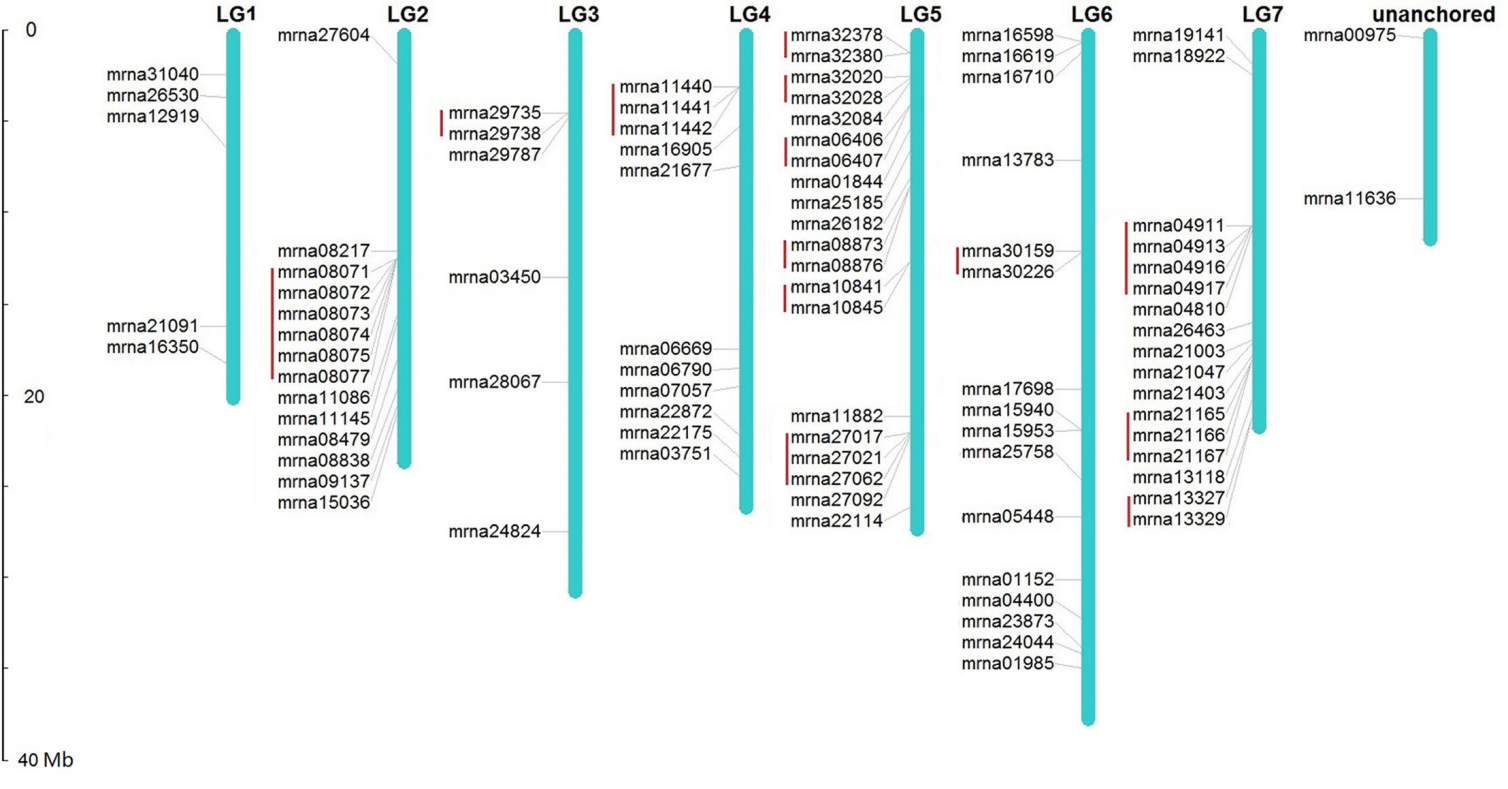
Locations of *FveERF* genes on the *Fragaria vesca* chromosomes. The size of a chromosome is indicated by its relative length. Tandemly duplicated genes are indicated with a red bar.

Chromosomal location analysis demonstrates that, except 2 *FveERF* genes found within unanchored chromosome sequences, the other 89 *FveERF*s are unevenly distributed among the seven *F. vesca* chromosomes (**Figure 1**). The number of *FveERF* genes on each chromosome has little relationship with chromosome length (correlation coefficient = 0.24), but is positively correlated with the number of tandem-arrayed *FveERF*s (correlation coefficient = 0.90). For example, LG5 and LG7, the two chromosomes with the largest numbers of *FveERF* genes (20 and 17, respectively), also contain the largest numbers of tandem *FveERF*s (13 and 9, respectively), whereas LG1 has the least number of *FveERF* genes (five) and has no tandem ones. This indicates that the uneven distribution of *FveERF*s is mainly due to the location of their tandem members. In total, 38.5% (35/91) of *FveERF* genes are arrayed in tandem repeats, strongly suggesting that a high proportion of *FveERF* genes are derived from tandem duplication events.

### Expansion of the FveERF Gene Family

**Figure 2:**
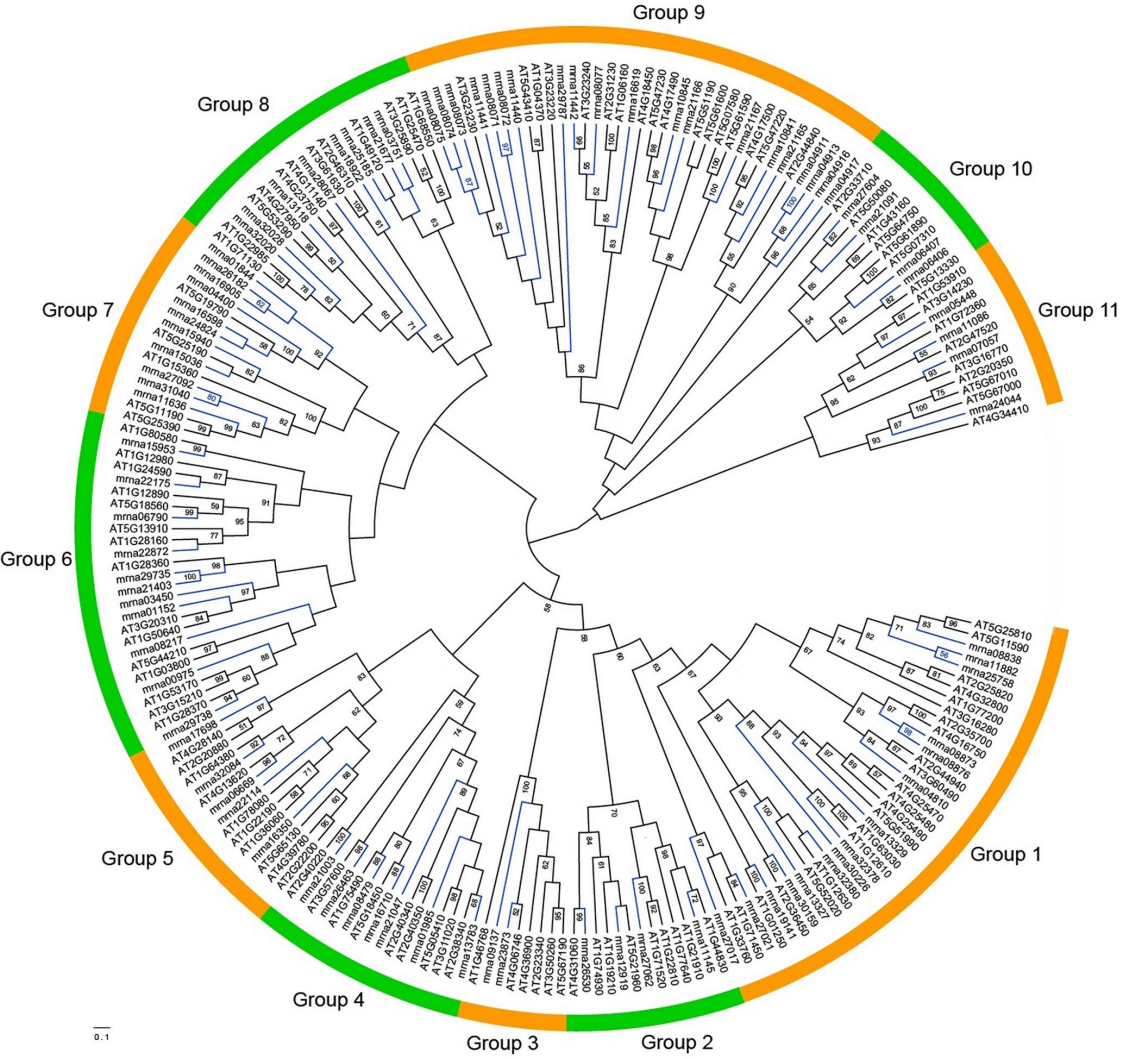
Maximum-likelihood phylogeny of the ERF proteins from *Fragaria vesca* and *Arabidopsis thaliana*. The phylogeny was constructed based on the amino acid sequences of full-length FveERF and AtERF proteins with 100 bootstrapping replicates. Bootstrap values greater than 50 are indicated on the nodes. Green and orange arcs indicate different groups of ERF proteins. Blue and black branches represent FveERF and AtERF proteins, respectively.

To study the relationships among *FveERF* genes, phylogenetic trees were constructed based on the ClustalX and MAFFT alignments of full-length FveERF and AtERF protein sequences, using ML (**Figure 2**) and aBayes (**Figure S2**) methods, respectively. All the phylogenies display similar grouping of the FveERF gene family, which is generally in consistence with the classification of *Arabidopsis ERF* genes (Nakano et al., 2006; **Table S2**). We further classified the *FveERF* genes of the 11 groups (groups 1–11) into two types: I) *FveERF*s that form phylogenetic clusters with other *FveERF*s and II) those that do not form clusters with other *FveERF*s but group with *AtERF* or *AtERF* and *FveERF* gene branch(es) (**Table S3**). The clustering of the type I *FveERF*s is likely a result of lineage-specific expansions of these genes in *F. vesca*. In contrast, type II *FveERF* genes are likely direct descendants of the ancestral genes in the common ancestor of *A. thaliana* and *F. vesca* and remain as single copies in the *F. vesca* genome. Among the 91 *FveERF*s, 24 genes, which form 10 phylogenetic clusters, belong to type I, and the remaining 67 genes belong to type II. This suggests that about one quarter of the *FveERF*s are involved in the expansions specific to the *Fragaria* lineage, while the rest three quarters likely have not expanded following the split of *Arabidopsis* and *Fragaria* lineages.

Chromosome location of the type I *FveERF*s shows that 11 (45.8%) of the 24 lineage-specific expanded *FveERF* genes are arrayed in tandem with their phylogenetically clustered genes. For instance, mrna08071–mrna08075 that form two clusters in group 9 of the phylogeny (mrna08071 and mrna08072 for one cluster and mrna08073–mrna08075 for another, **Figures 2** and **S2**) are located in a six-gene tandem repeat on chromosome 2 (**Figure 1**). These genes are likely derived from tandem duplications, and are hereafter referred to as lineage-specific tandem *FveERF*s. However, not all the type I *FveERF*s located in tandem repeat are lineage-specific tandem *FveERF*s. For instance, the type I gene mrna29735 is phylogenetically clustered with mrna21403 (**Figures 2** and **S2**) but is arrayed in tandem with mrna29738 (**Figure 1**). The relationship among these three genes suggests that a tandem duplication gave rise to the gene pair mrna29735 and mrna29738 rather than the lineage-specific gene pair of mrna29735 and mrna21403. The MCScanX analysis indicates that, among the twelve non-tandem type I *FveERF*s, seven genes including mrna21403 likely are derived from dispersed duplications, while the rest five are likely from segmental duplications (**Table S3**). Collectively, tandem duplication is the major mechanism for the lineage-specific expansion of the *FveERF* gene family.

In addition to the type I lineage-specific tandem *FveERF*s, 23 (34.3%) of the 67 type II *FveERF* genes that have not undergone lineage-specific expansion also reside in tandem repeats on chromosomes (**Figure 1**, **Table S3**). For example, the type II *FveERF*s mrna10841 and mrna10845 in group 9 are located in a two-gene tandem repeat on chromosome 2. Interestingly, their phylogenetically clustered *AtERF* orthologs (AT5G47220 and AT4G17500 for mrna10841; AT5G47230 and AT4G17490 for mrna10845, **Figures 2** and **S2**) are also arrayed in tandem on *A. thaliana* chromosomes (AT5G47220 and AT5G47230; AT4G17500 and AT4G17490). Therefore, it is very likely that mrna10841 and mrna10845 are derived from ancestral tandem duplications in the most recent common ancestor of *A. thaliana* and *F. vesca* and are maintained in tandem following the split of the two lineages.

There are a total of 15 tandem type II *FveERF* genes having tandem *AtERF* orthologs (**Figures 2** and **S2**, **Table S3**), indicating they are derived from ancestral tandem repeats. Among them, two genes are tandemly arrayed with type I *FveERF* genes, i.e., mrna29738 tandem with mrna29735, and mrna08077 tandem with mrna08071–mrna08075 (**Figure 1**). This suggests that these type I genes are involved in both ancestral and lineage-specific tandem duplications. On the other hand, the rest 10 tandem type II *FveERF*s are phylogenetically clustered with their *AtERF* orthologs which are not arrayed in tandem. We still considered these 10 *FveERF*s to originate from ancestral tandem duplications, because the *A. thaliana* genome has undergone extensive chromosomal rearrangements (del Pozo and Ramirez-Parra, 2015) which would lead to non-tandem arrangements of *AtERF* orthologs. Therefore, at least 34.1% (31 of all 91) *FveERF* genes can be classified into ancestral tandem *FveERF*s.

Taken together, we define the tandem *FveERF* genes that cluster with each other in the phylogenies as lineage-specific tandem *FveERF*s, while the tandem *FveERF*s phylogenetically clustering with their *AtERF* orthologs or retaining in singletons as ancestral tandem *FveERF*s. From the above analyses, the total 35 tandem *FveERF*s include 11 lineage-specific ones and 29 ancestral ones, with 5 belonging to both.

### Motif and Gene Structures of *FveERF* Genes

**Figure 3:**
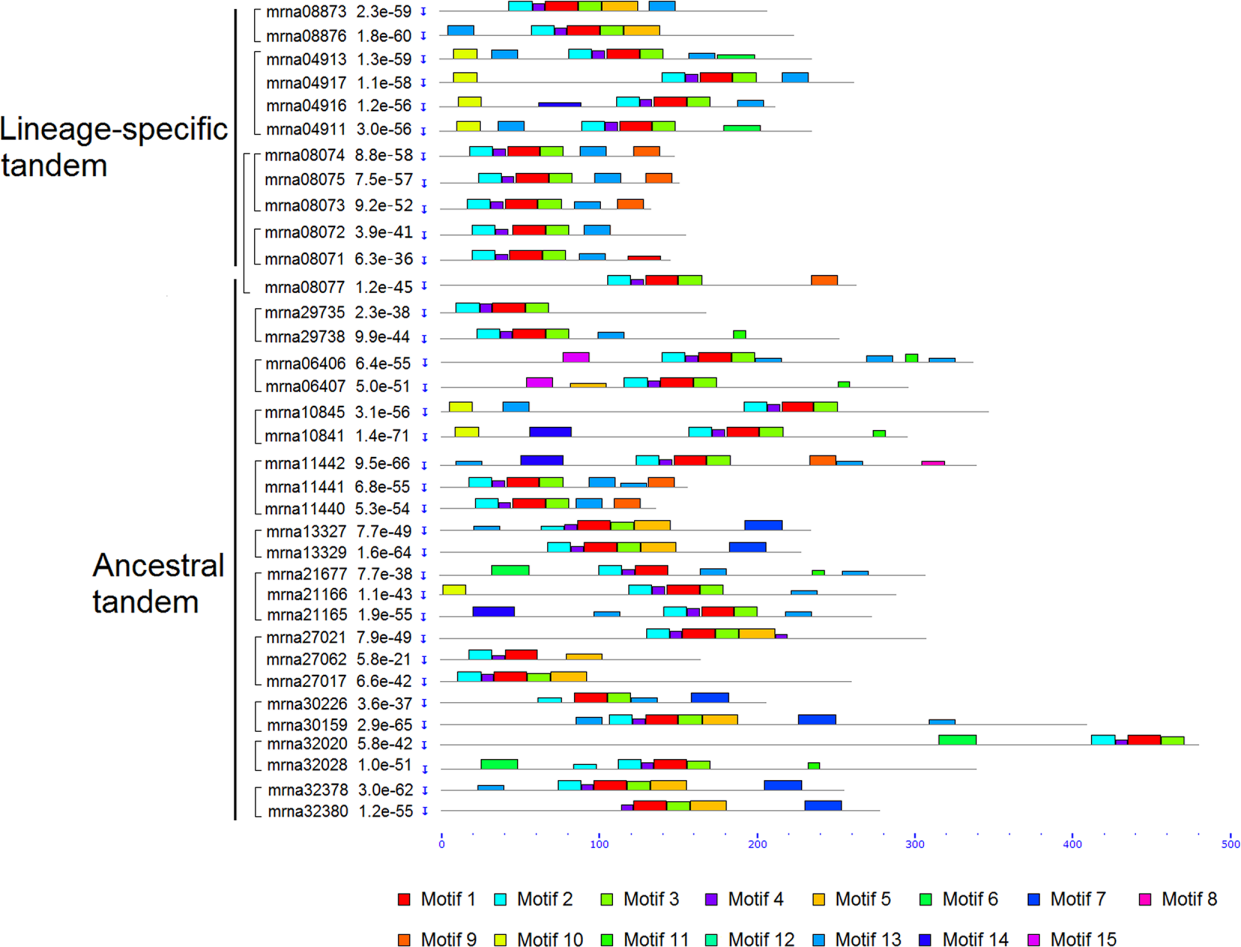
Schematic diagram of amino acid motifs of tandem FveERF proteins. Motif analysis was performed using MEME5.0.1 as described in the *Materials and Methods*. Proteins whose genes located in the same tandem repeat are grouped together. Mrna08077 forms an ancestral tandem repeat with mrna08071–mrna08075.

We analyzed motif structures of the FveERF proteins, with 15 conserved motifs (motifs 1–15) identified using MEME suite (**Figures 3**, **S3**, and **S4**). Motifs 1–4 correspond to the AP2/ERF domain and have been identified in nearly all FveERF proteins. The four lineage-specific tandem FveERF pairs show differences in the arrangement of zero to four motifs (an average of 1.75), where totally only four motifs have been differentially identified. In contrast, the ancestral tandem pairs have differences in 1–6 (an average of 3.26) of the 12 differentially distributed motifs, which include motifs 1–4 that are key to the AP2/ERF domain. The average number of *FveERFs* in an ancestral or lineage-specific tandem repeat is similar (2.5 for ancestral vs. 2.75 for lineage-specific tandem repeat). However, the motif analysis demonstrates that the protein structure of the ancestral tandem *FveERFs* is more divergent than that of the lineage-specific tandem ones.

With respect to gene structure, 24 (26.4%) of the 91 *FveERF* genes possess introns (**Table S3**). The average number of introns per intron-containing *FveERF* is 1.83. Around half of these genes (13 of 24) contain a single intron, with others contain two to three except for one that contains eight. These intron-containing *FveERF*s are located on chromosomes 1–6 as well as the unanchored scaffold (**Table S3**). None are found on chromosome 7, which houses the second-most (17) *FveERF* genes. All genes within the four lineage-specific tandem *FveERF* pairs have same numbers of introns with their counterparts, whereas in about half of the 11 ancestral tandem *FveERF* pairs exon/intron structures are different, indicating that the gene structures of ancestral tandem *FveERF*s have diverged.

### Expression Profiles of *FveERF* Genes in Flowers and Fruits

**Figure 4:**
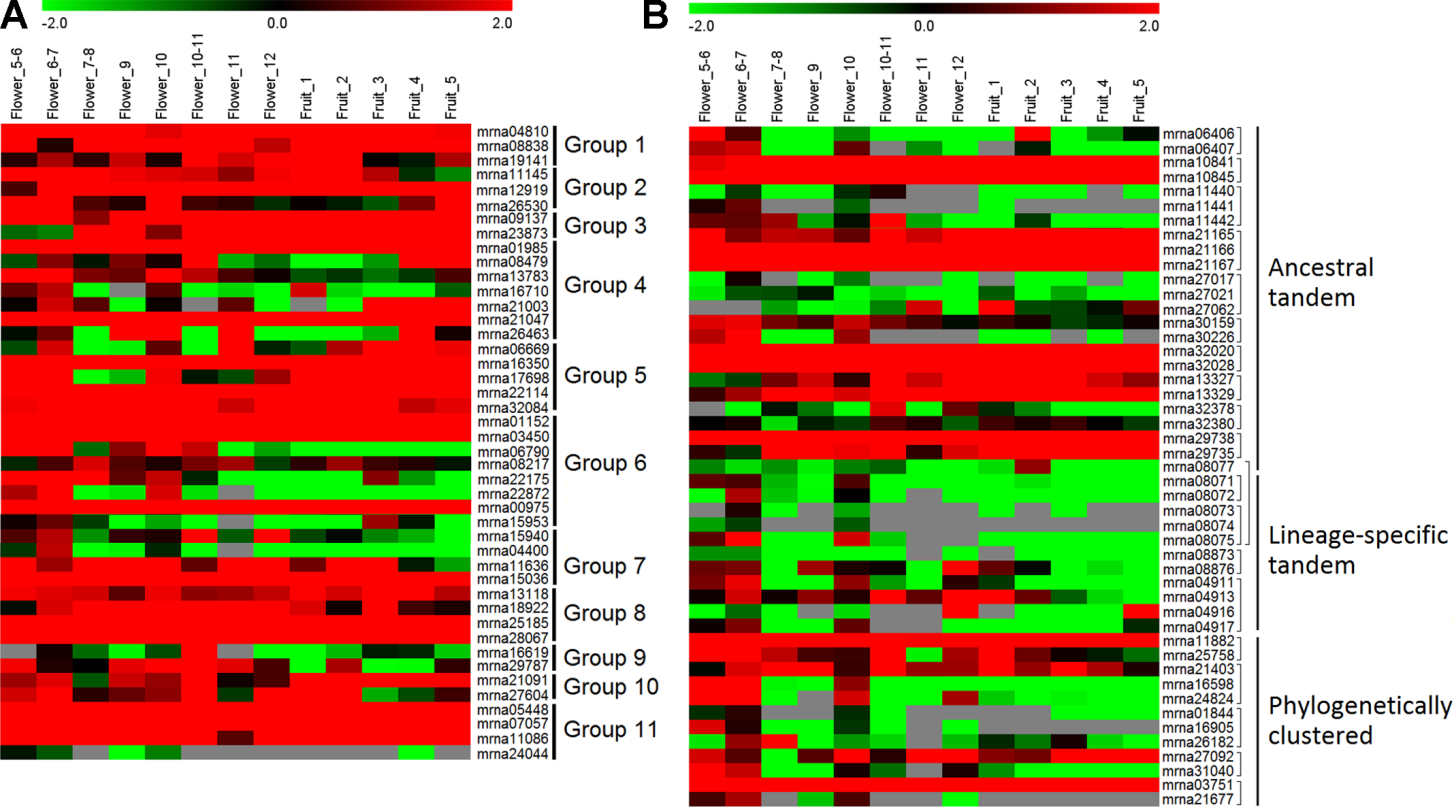
Expression profiles of *FveERF* genes in different stages of *Fragaria vesca* flowers and early-stage fruits. **(A** and **B)** The mRNA levels of the non-tandem **(A)** and tandem/phylogenetically clustered **(B)** *FveERF* genes. Genes located in the same tandem repeat or in a phylogenetic cluster are grouped together. Mrna08077 forms an ancestral tandem repeat with mrna08071–mrna08075. Mrna21403 forms a phylogenetic cluster with mrna29735. Data were retrieved from http://bioinformatics.towson.edu/strawberry/ (Hollender et al., 2012; Darwish et al., 2013; Kang et al., 2013). Expression levels were calculated in the log2 scale. For a detailed description of the stages, please see http://bioinformatics.towson.edu/strawberry/newpage/Tissue_Description.aspx.

To investigate the expression profiles of *FveERF* genes, we downloaded and analyzed the transcriptomic data of *F. vesca* flowers and early fruits (Hollender et al., 2012; Darwish et al., 2013; Kang et al., 2013). All the *FveERF* genes have RPKM values larger than 0.3 in at least two flower-development stages (**Figure 4**); thus, we consider all *FveERF*s to be expressed during flower development in *F. vesca* (see *Materials and Methods*). In contrast, RPKM values for 18 (19.8%) *FveERF*s are lower than 0.3 throughout early-stage fruit development. The expression levels of *FveERF*s in tissues of flowers and early fruits (**Figure S5**) are similar to those in the stages. These results indicate that most, if not all, *FveERF* genes are involved in flower development, whereas ∼20% of *FveERF*s may not participate during early-stage fruit development.

The expression levels of tandem or phylogenetically clustered genes are significantly different from those of the non-tandem/clustered *FveERF*s (all *p* < 0.001 from *t*-test). Moreover, among the 33 *FveERF*s with low expression levels (RPKM values <1 in at least two thirds of the 13 stages of reproductive development, **Figure 4**), 81.8% (27) either cluster on the phylogeny or are arrayed in tandem on chromosomes. Meanwhile, 60.1% of the 47 tandem or clustered *FveERFs* have low expression levels, 4.4-fold higher than the percentage of low-expression genes among the other 44 *FveERF*s (13.6%). This percentage increases to 81.8% (9 of 11) for lineage-specific tandem *FveERF*s, 0.8-fold higher than for ancestral tandem *FveERF*s (45.8%). Consistently, the expression levels of lineage-specific tandem *FveERF*s are also significantly lower than those of the ancestral ones (*p* < 0.001). These results demonstrate that the expression levels of tandem or clustered *FveERF*s are lower than those of the other *FveERF*s during reproductive development, with lineage-specific tandem *FveERF*s having the lowest expression.

The expression patterns of tandem *FveERF* pairs are less diversified than those of the non-tandem ones in a same group in flowers and early fruits (**Figures 4** and **S5**). More than 75% non-tandem *FveERF* gene pairs in a group show diversified expression patterns (data not shown), while approximately 50% of tandem *FveERF* pairs have positive correlated expression patterns (correlation coefficient >0.5, **Table S4**). Further, this percentage is nearly the same for both the ancestral tandem FveERF gene pairs and the lineage-specific tandem ones. This suggests that the expression patterns of ancestral and lineage-specific tandem *FveERF* duplicates diverge to similar degrees in flowers and early-stage fruits, regardless of the increased age and evolutionary history of ancestral duplicates.

**Figure 5:**
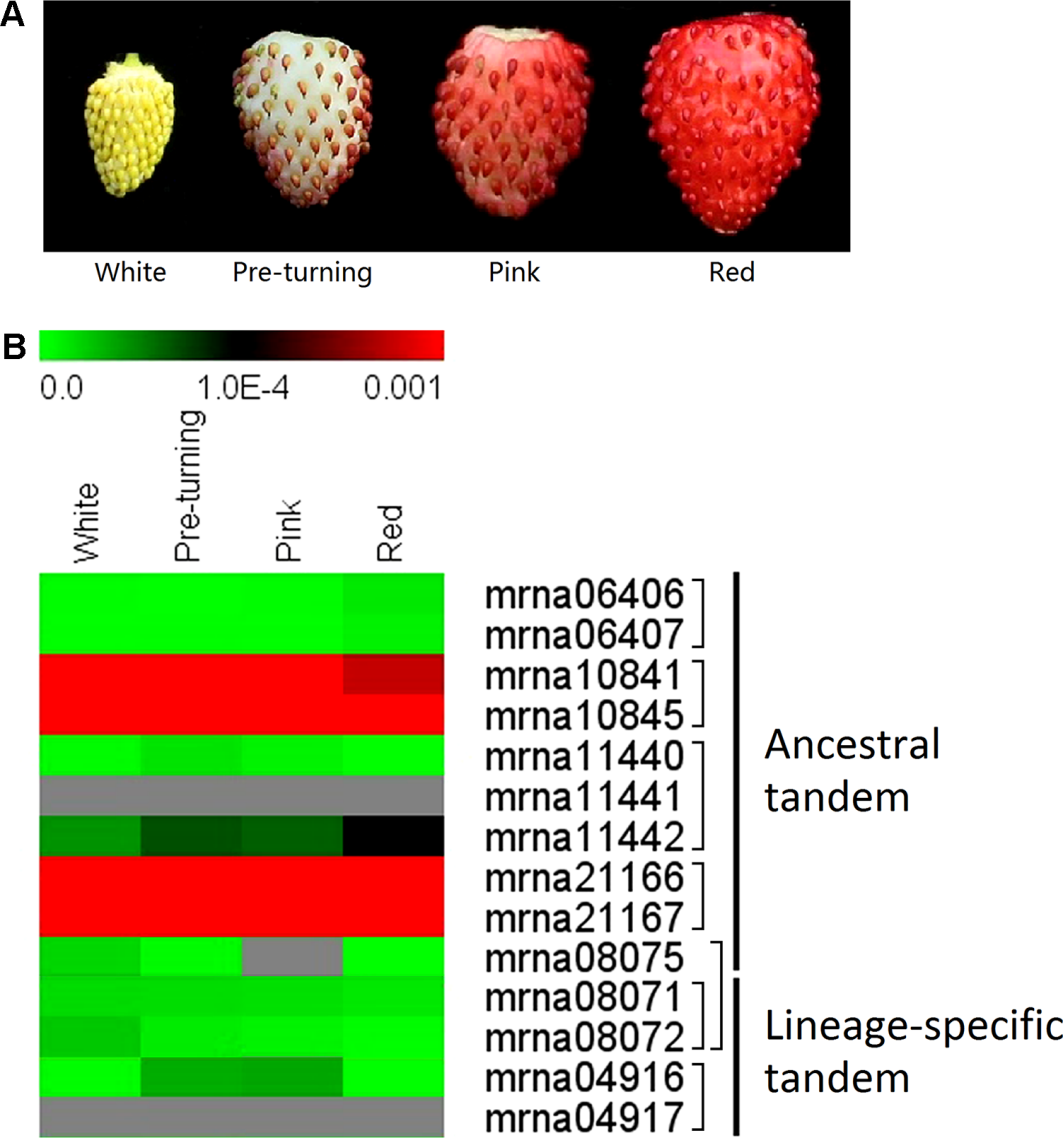
Expression profiles of tandem *FveERF* genes during fruit ripening. **(A)** The schematic diagram for the four stages of fleshy fruits investigated in B. **(B)** The expression levels of tandem *FveERF* genes relative to *GAPDH*, measured by quantitative RT-PCR and displayed in the log2 scale. Genes located in the same tandem repeat are grouped together. Mrna08075 forms an ancestral tandem repeat with mrna08071 and mrna08072. Three biological replicates and three technical replicates were obtained for each data point.

We further investigated expression patterns of the ancestral and lineage-specific tandem *FveERF*s (see *Materials and Methods* for the selection of the tandem *FveERF*s) during the fruit-ripening stages of *F. vesca* using qRT-PCR (**Figure 5A**). The five lineage-specific tandem *FveERF*s have very low expression (< 1 × 10^−4^ when using *FveGAPDH* as the reference gene) throughout the ripening stages. Five of the nine ancestral tandem *FveERF*s have no detectable expression during these stages, whereas the remaining four (found within two tandem repeats) exhibit much higher expression (**Figure 5B**). These expression patterns are roughly in accordance with the expression patterns for *FveERF*s in early fruits (**Figures 4** and **5B**). Therefore, the tandem *FveERF* genes are most likely consistently expressed throughout fruit development and ripening stages.

### Expression of Tandem Duplicated *FveERF* Genes Under Drought/Cold Stress

**Figure 6:**
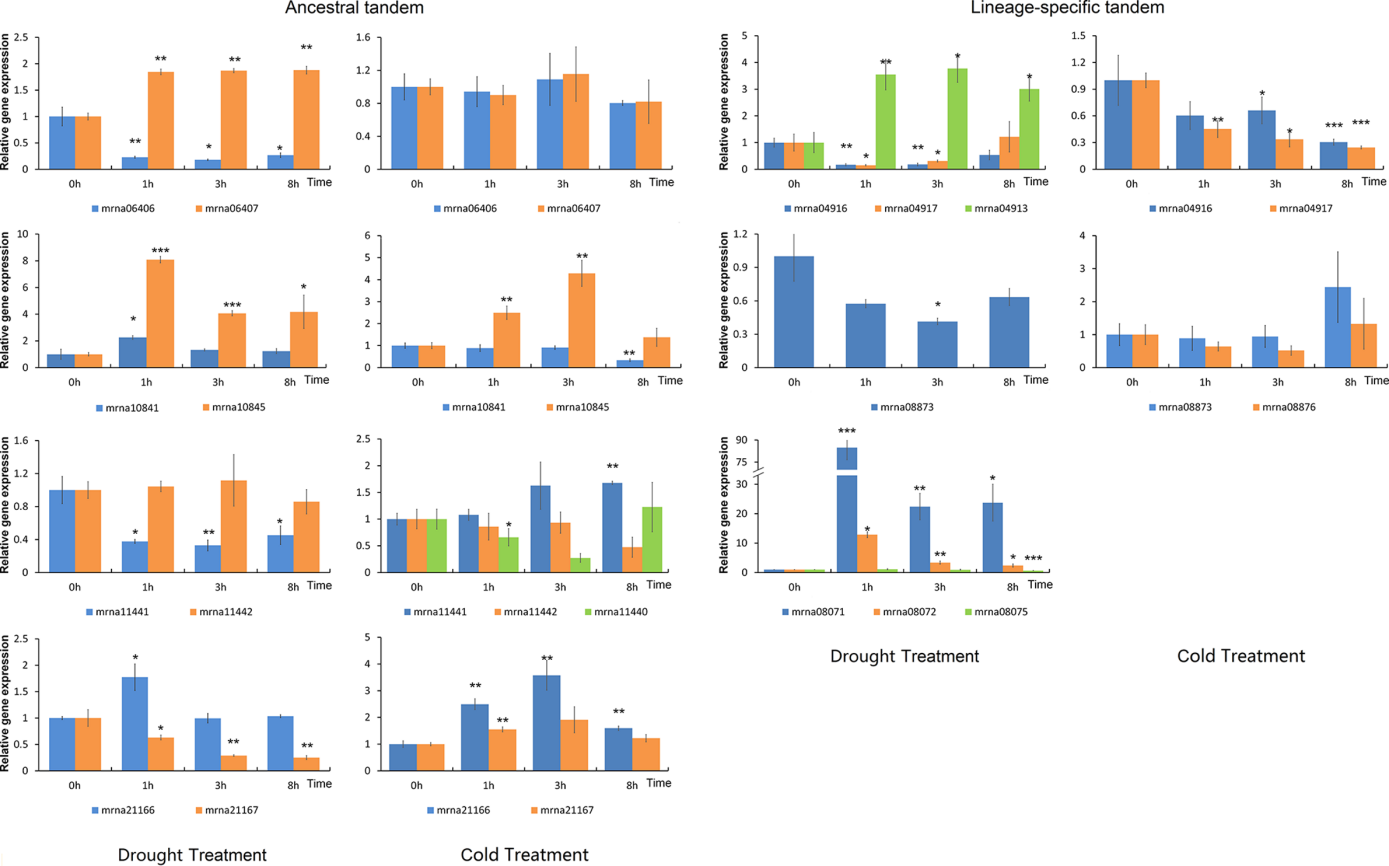
Expression profiles of *FveERF* genes in response to drought and cold. The expression levels relative to *GAPDH* were measured by quantitative RT-PCR. Three biological replicates and three technical replicates were obtained for each data point. Asterisks above the error bars indicate significant differences between the treated and untreated (0h) samples (*p < 0.05; **p < 0.01; ***p < 0.001). Mrna08075 forms an ancestral tandem repeat with mrna08071 and mrna08072. The genes with expression levels lower than 1 × 10^−4^ at most time points of the treatment are not shown.

ERF transcription factors play important roles in abiotic stress response (Lata and Prasad, 2011). We treated the *F. vesca* seedlings with either cold or drought stress, and characterized the expression of nine lineage-specific and nine ancestral tandem *FveERF*s (see *Materials and Methods* for the selection of the tandem *FveERF*s, **Figure 6**). Similar to in fruits, lineage-specific tandem *FveERF*s have very low expression levels in *F. vesca* seedlings, regardless of treatment (**Table S5**). Six of the nine lineage-specific tandem *FveERF*s (mrna04911, mrna04913, mrna08071, mrna08072, mrna08075 and mrna08876) have no detectable gene expression under either or both stresses, while only one ancestral tandem *FveERF* (mrna11440) is undetectable under drought stress. Further, among the expressed *FveERF*s, the average expression level of ancestral tandem ones is approximately 100-fold higher than that of the lineage-specific tandem ones (**Figure 5B**). These results suggest that *FveERF* genes generated by recent tandem duplications may generally have low expression levels.

We have observed that the ancestral tandem *FveERF* pairs are differentially expressed following stress treatment, cold or drought (**Figure 6** and **Table S6**). The ancestral tandem pair of mrna11440, mrna11441, and mrna11442 displays divergent expression patterns following both stress treatments, while the other three pairs are only differentially expressed following either cold or drought stress. In contrast, all lineage-specific tandem *FveERF* pairs exhibit similar stress-response expression patterns, except for mrna04913 (compared to mrna04916 or mrna04917) following dehydration (**Figure 6** and **Table S6**). Based on these data, *FveERF* duplicates from ancestral tandem duplications seem to have diverged in their responses to abiotic stress, whereas most lineage-specific tandem genes have not.

## Discussion

**Figure 7:**
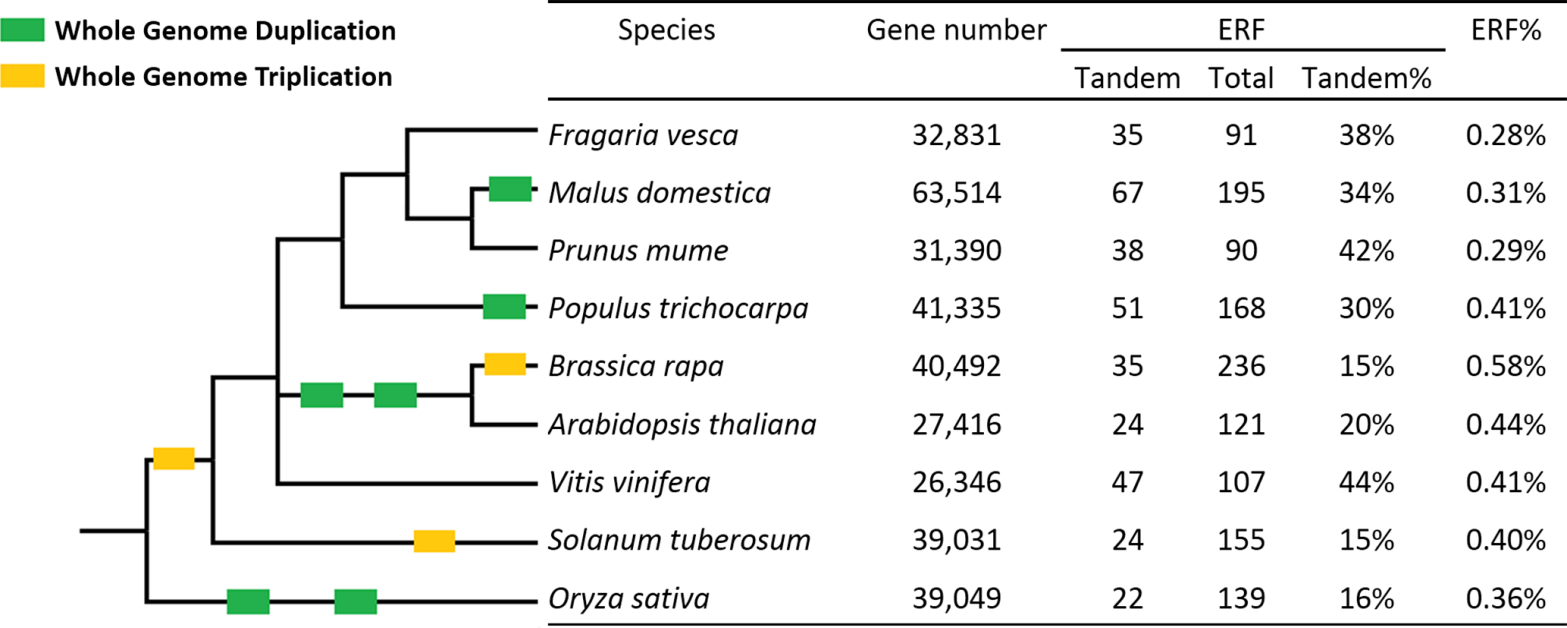
Percentages of tandem *ERF* genes in the nine species investigated. ERF% shows the percentage of *ERF* genes in the total gene set. The Taxonomy Common Tree constructed online by Taxonomy Browser in the National Center for Biotechnology Information (NCBI; http://www.ncbi.nlm.nih.gov/Taxonomy/CommonTree/wwwcmt.cgi) is on the left. The branch length is not proportional to the evolutionary time. Green box, whole-genome duplication; yellow box, whole-genome triplication.

This is the first study identifying *ERF* genes in woodland strawberry (*F. vesca*). A total of 91 *FveERF*s have been identified and divided into 11 groups based on phylogenetic and motif analyses. The percentage of *ERF* genes in total protein-coding genes in *F. vesca* (0.28%, **Figure 7**) is similar to the percentages found in two other Rosaceae family plants, plum [*Prunus mume*, 0.29% (Du et al., 2013)] and apple [*Malus × domestica*, 0.31% (Zhuang et al., 2011)], but lower than those in Brassicaceae family species, such as *A. thaliana* [0.44% (Nakano et al., 2006)] and *Brassica rapa* [0.58% (Song et al., 2013)]. The higher percentage of *AtERF* genes is likely a result of the polyploidization events during the evolution of *A. thaliana*, as 75% of them are proposed to have been preferentially retained after WGDs (Nakano et al., 2006). As being transcription factor genes, *ERF*s would have been retained at a higher than average level after WGD, but not after tandem duplication (Panchy et al., 2016). However, the apple genome that has undergone a recent WGD event does not contain higher percentage of *ERF* genes than *F. vesca*. Our results demonstrate that more *FveERF* genes are involved in tandem duplication than in WGD/segmental duplication, suggesting that tandem duplication is the major mechanism contributing to the expansion of the *FveERF* gene family.

The percentage of tandem *FveERF*s in total *FveERF*s is similar to that of *PmuERF*s in plum and of *VvERF*s in grapevine (*Vitis vinifera*), a little higher than that in apple and poplar (*Populus trichocarpa*), and much higher than that in *A. thaliana* and *B. rapa* (**Figure 7**). *F. vesca*, plum and grapevine have not undergone any WGDs after the triplication event (γ) probably shared by all core eudicots (Bowers et al., 2003; Jaillon et al., 2007; Cenci et al., 2010), while apple and poplar have undergone WGD once (Tuskan et al., 2006; Velasco et al., 2010) and *A. thaliana* and *B. rapa* have undergone WGD at least twice (Bowers et al., 2003). Therefore, the percentage of tandem *ERF* genes retained seems to be negatively correlated with occurrences of the polyploidization events, possibly because of the rearrangement of chromosomal sequences after WGD.

The higher percentage of tandem *ERF* genes in *F. vesca* than in *A. thaliana* is mainly due to a greater number of ancestral tandem *ERF*s (31 vs. 17), rather than lineage-specific tandem ones (11 vs. 11). Further, all ancestral tandem *AtERF*s have tandem *FveERF* orthologs, whereas there are 10 ancestral tandem *FveERF*s whose *AtERF* orthologs are not arrayed in tandem. This number difference of tandem orthologs suggests that the more ancestral tandem *ERF* genes in *F. vesca* than in *A. thaliana* are due to more rearrangements or losses of the ancestral tandem *AtERF*s. Extensive rearrangement and loss of chromosomal segments have occurred in *A. thaliana* during its rediploidization after polyploidization (del Pozo and Ramirez-Parra, 2015). Ancestral tandem *AtERF*s are defined as those derived from tandem duplications in the common ancestor of *F. vesca* and *A. thaliana*, which occurred prior to the twice polyploidization of the *Arabidopsis* lineage. Hence, the ancestral tandem *AtERF*s have experienced at least once rediploidization, leading to the number difference of ancestral tandem *ERF* genes between *F. vesca* and *A. thaliana*. Altogether, genomic rearrangement during rediploidization following polyploidization is an important factor affecting the retention of ancestral tandem *ERF* genes. The higher retention of tandem *FveERF*s than tandem *AtERF*s may be largely attributed to no polyploidization occurred in *F. vesca* after the divergence of core eudicots.

The discrimination of ancestral and lineage-specific tandem *FveERF* genes provides us with a good tool to compare the divergence of tandem *FveERF* duplicates generated at different times. As expected, the average values of pairwise nucleotide divergence, synonymous nucleotide substitutions per synonymous site (Ks), and non-synonymous substitutions per nonsynonymous site (Ka) between lineage-specific tandem *FveERF* pairs are significantly lower than those between ancestral tandem *FveERF* pairs, respectively (**Table S7**). Moreover, lineage-specific tandem *FveERF* genes maintain higher similarities of exon/intron and motif structures than the ancestral tandem ones. These results indicate that sequence and structure divergences of ancestral tandem *FveERF*s are higher than those of lineage-specific tandem *FveERF*s. None of the ancestral tandem *AtERF*s contain an intron (Nakano et al., 2006). In contrast, 35.5% (11 of 37) ancestral tandem *FveERF*s have an average number of 2.36 introns. Particularly, half of ancestral tandem *FveERF* pairs show variable exon/intron structures. Thus, it seems that intron gain/loss has occurred more frequently in the evolutionary histories of *FveERF* genes compared to *AtERF*s, which may play a role in the divergence of *FveERF*s, especially for ancestral tandem ones.

Tandem duplicates are proposed to have higher expression correlation than the duplicates derived from most of the other mechanisms (Wang et al., 2012b). However, our analyses show that the expression correlation of lineage-specific tandem *FveERF*s in flowers and fruits is lower than that of other lineage-specific expanded *FveERF*s, but is similar to that of the ancestral tandem ones (**Table S4**). The studies on expression patterns of tandem duplicates in other families, such as the C_2_H_2_ zinc-finger gene family in rice (Agarwal et al., 2007) and the phosphatidylethanolamine binding protein (PEBP) family in soybean (Wang et al., 2015), also demonstrate that ancestral and lineage-specific tandem duplicates have similarly highly diversified expression patterns in developmental tissues. These results support that expression of tandem *FveERF* duplicates in reproductive development has diverged shortly after duplication.

Previous studies have suggested that expression divergence of the tandem duplicates has little relationship with their Ks values (Ganko et al., 2007), mainly based on expression analyses in developmental tissues/organs. Our results with respect to tandem *FveERF* expression in reproductive development are consistent with this suggestion. However, the results under stressed conditions show different patterns. All expressed lineage-specific tandem *FveERF* duplicates exhibit same response patterns upon drought or cold treatment with only one exception, whereas the ancestral ones diverge at a much higher level (**Table S6**). This suggest that expression divergence of tandem *FveERF*s under stress may have occurred later, but evolved faster, than in reproductive development. In addition to growth and development, *ERF*s are also important in the regulation of abiotic stress responses in plants (Lata and Prasad, 2011). Although the roles of the sampled tandem *FveERF*s in abiotic stress responses have not been revealed so far, the *A. thaliana* groups containing their *AtERF* orthologs have been shown with functions in tolerance to abiotic stress. Moreover, the tandem *FveERF*s show induced or reduced expression after drought and cold treatments, supporting that they likely play roles in the responses to these stresses. Therefore, the high expression divergence of the ancestral tandem *FveERF*s under stress conditions could contribute to the responses of *F. vesca* to abiotic stresses.

Besides, with respect to expression levels, no matter under stress conditions or in reproductive development, high proportions of lineage-specific tandem *FveERF* pairs are undetectable. Comparatively, all ancestral tandem *FveERF* pairs, at least one of the members, are expressed at much higher levels. Expression levels of the ancestors of the undetectable lineage-specific tandem *FveERF*s are unknown; analyses on their orthologs in *A. thaliana* and other plants may provide indication that whether recent tandem duplication is a main cause of such low expression levels of these lineage-specific tandem *FveERF* pairs. On the other hand, like in expression patterns, the divergence in expression levels of the expressed lineage-specific tandem *FveERF*s is at similar levels with the ancestral tandem ones in flower and fruit stages, but lower under abiotic stressed conditions (**Table S5**). Thus, the expression divergence of tandem *FveERF* duplicates is probably slower under stress conditions than in reproductive development at early stage after the duplication.

## Conclusions

In this study, the ERF gene family in *F. vesca* was identified and analyzed, especially for their tandem members. Compared with ancestral tandem *FveERF*s, the lineage-specific tandem *FveERF*s are more conserved in sequence, structure, and expression under abiotic stress, whereas are similarly highly diversified in expression during reproductive development. These results suggest that the retention of tandem *FveERF* duplicates soon after their duplication may be related to their divergence in the regulation of reproductive development. On the other hand, their further divergence in response patterns to abiotic stresses likely contributes to stress responses of *F. vesca*. This provides new insights into the expression divergence between tandem duplicates in plants.

## Author Contributions

YL and JD designed the experiments. XW and SL performed the experiments and data analyses. DL and QW participated in data analyses. JD, XW, and SL wrote the manuscript. YL and RM revised the manuscript. All authors read and approved the final manuscript.

## Funding

This work was supported by the National Natural Science Foundation of China Grants 31471860 (to JD and YL), and A Project Funded by the Priority Academic Program Development of Jiangsu Higher Education Institutions (to YL).

## Conflict of Interest Statement

The authors declare that the research was conducted in the absence of any commercial or financial relationships that could be construed as a potential conflict of interest.
