# Supplementary material for: Characterization and Expression Analysis of ERF Genes in Fragaria vesca Suggest Different Divergences of Tandem ERF Duplicates
Source: Front Genet. 2019 Sep 12;10:805. doi: 10.3389/fgene.2019.00805 (PMC6752658; doi:10.3389/fgene.2019.00805)
Supplement: Supplementary file 1 [file DataSheet_1.pdf]

Electronic Supplementary Material

Article title: Characterization and expression analysis of *ERF* Genes in *Fragaria vesca* suggest different divergences of tandem *ERF* duplicates

Journal name: Frontier in Genetics

Author names: Xiaojing Wang. Shanshan Lin. Decai Liu. Quanzhi Wang. Richard McAvoy. Jing Ding. Yi Li

Corresponding authors:

Jing Ding

State Key Laboratory of Crop Genetics and Germplasm Enhancement and College of Horticulture, Nanjing Agricultural University, Nanjing, P. R. China; jding@njau.edu.cn.

Yi Li

State Key Laboratory of Crop Genetics and Germplasm Enhancement and College of Horticulture, Nanjing Agricultural University, Nanjing, P. R. China; Department of Plant Science and Landscape Architecture, University of Connecticut, Storrs, CT, USA (Yi Li holds a 2-month/year visiting professor position at Nanjing Agricultural University); yi.li@uconn.edu.

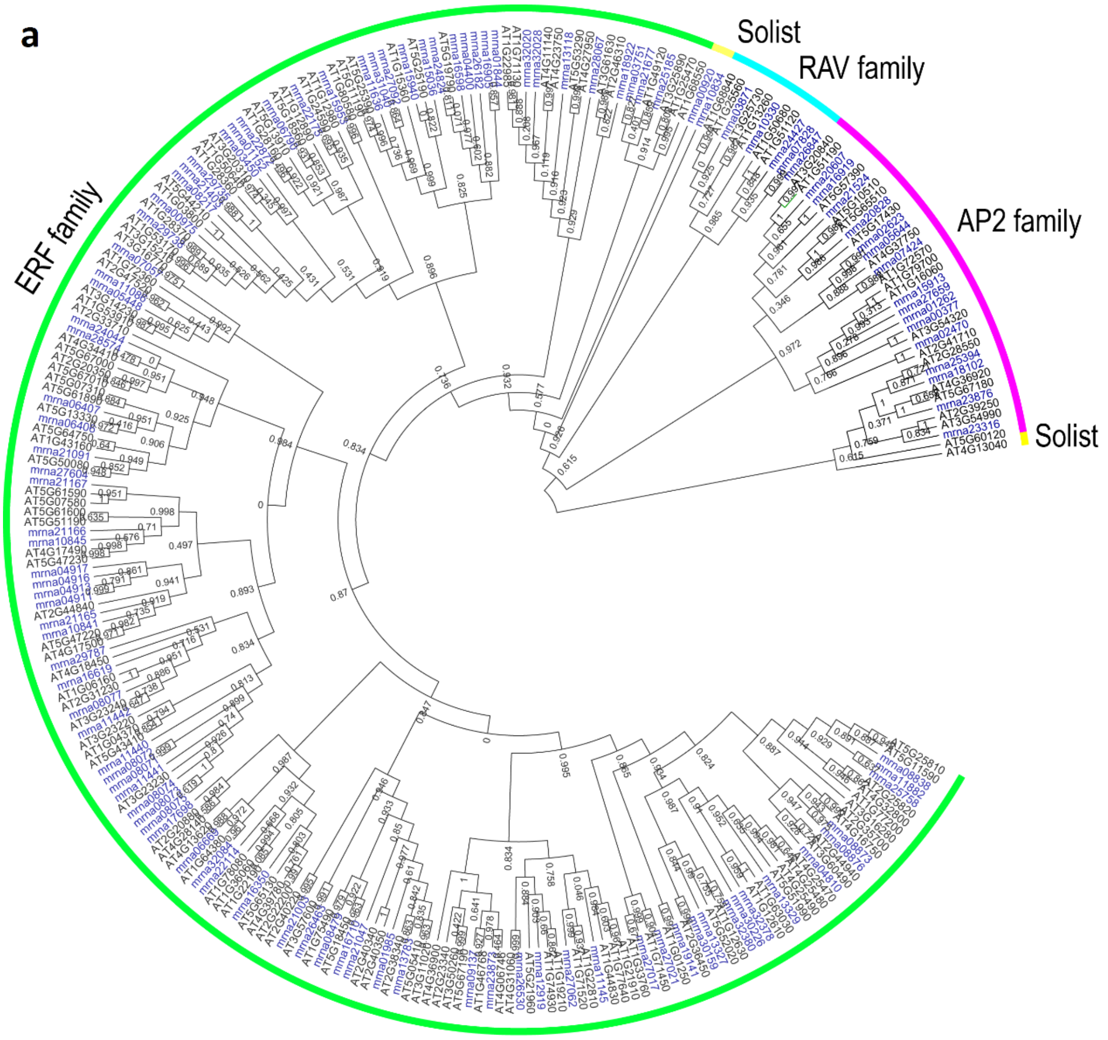
a
ERF family
Solist
RAV family
AP2 family
Solist

b

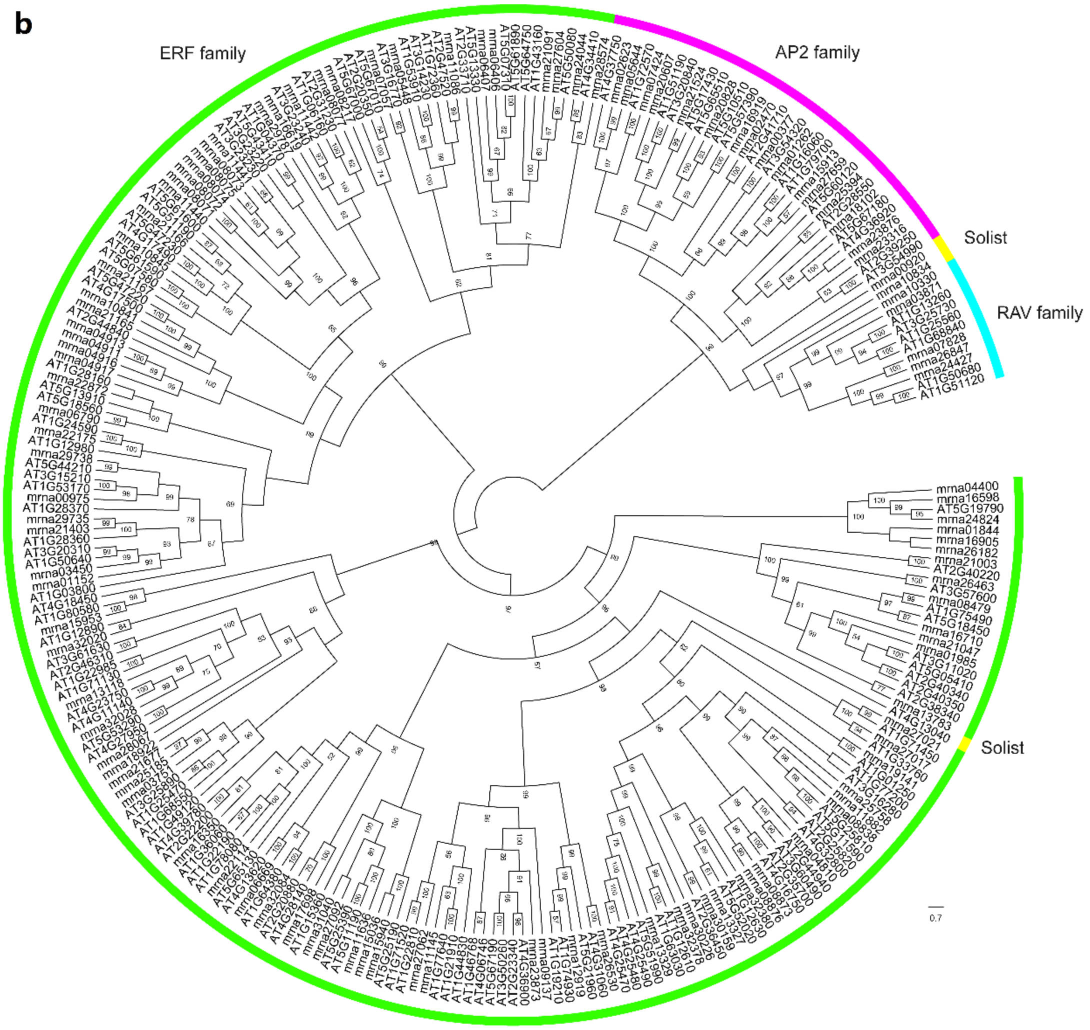

Fig. S1 Phylogenetic analyses of the AP2/ERF superfamily genes from *F. vesca* and *A. thaliana*. **a** ~~Maximum-likelihood phylogeny based on the ClustalX alignment of the full-length AP2/ERF protein sequences with 100 bootstrapping replicates using PhyML 3.0. Bootstrap values greater than 50 are indicated on the nodes~~. **b** Unrooted aBayes phylogeny based on the MAFFT alignment of the full-length AP2/ERF protein sequences. Bayes posterior probabilities are given for branches. Green, light blue, pink, and yellow arcs represent ERF, RAV, AP2 and Solist, respectively.

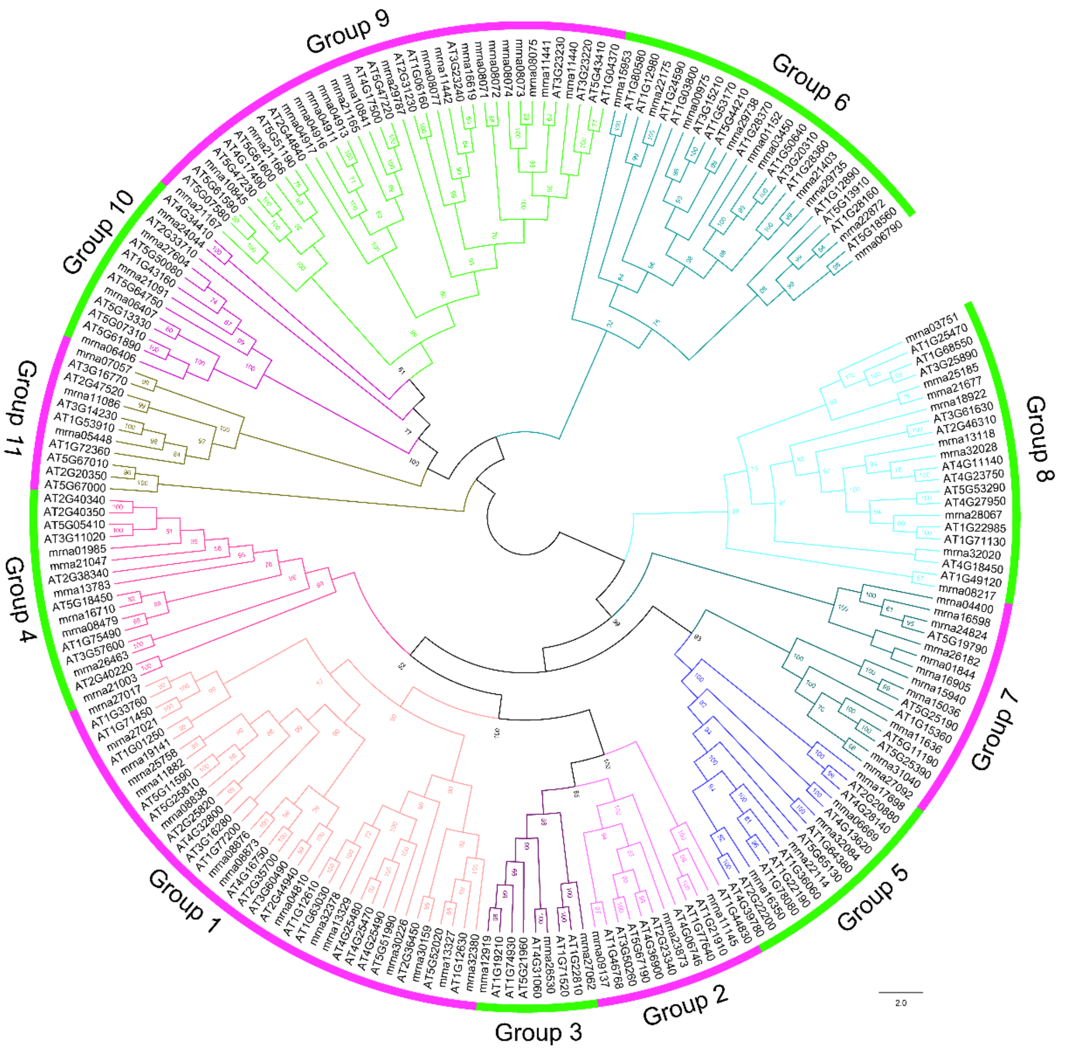

Fig. S2 Unrooted tree of the putative ERF protein sequences from *F. vesca* and *A. thaliana* obtained by the aBayes method based on the alignment using MAFFT v7. Bayes posterior probabilities (>50) are given for branches. Green and orange arcs indicate different groups of ERF proteins.

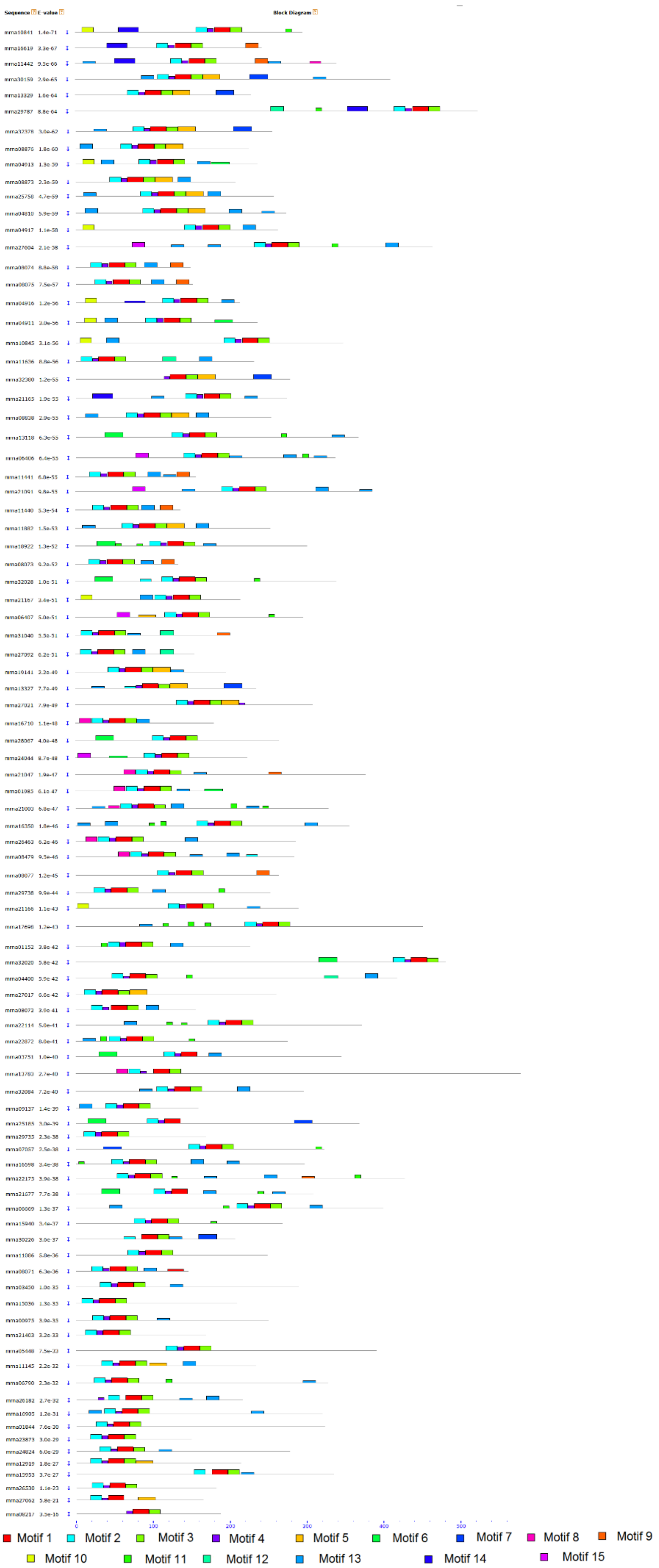
Sequence
E value
Block Diagram
Motif 1
Motif 2
Motif 3
Motif 4
Motif 5
Motif 6
Motif 7
Motif 8
Motif 9
Motif 10
Motif 11
Motif 12
Motif 13
Motif 14
Motif 15

Fig. S3 Schematic diagram of amino acid motifs of all FveERF proteins. Motif analysis was performed using MEME5.0.1 as described in the methods. Fifteen motifs (1 to 15) were identified and indicated by different colors. Motif location and combined p-value are showed.

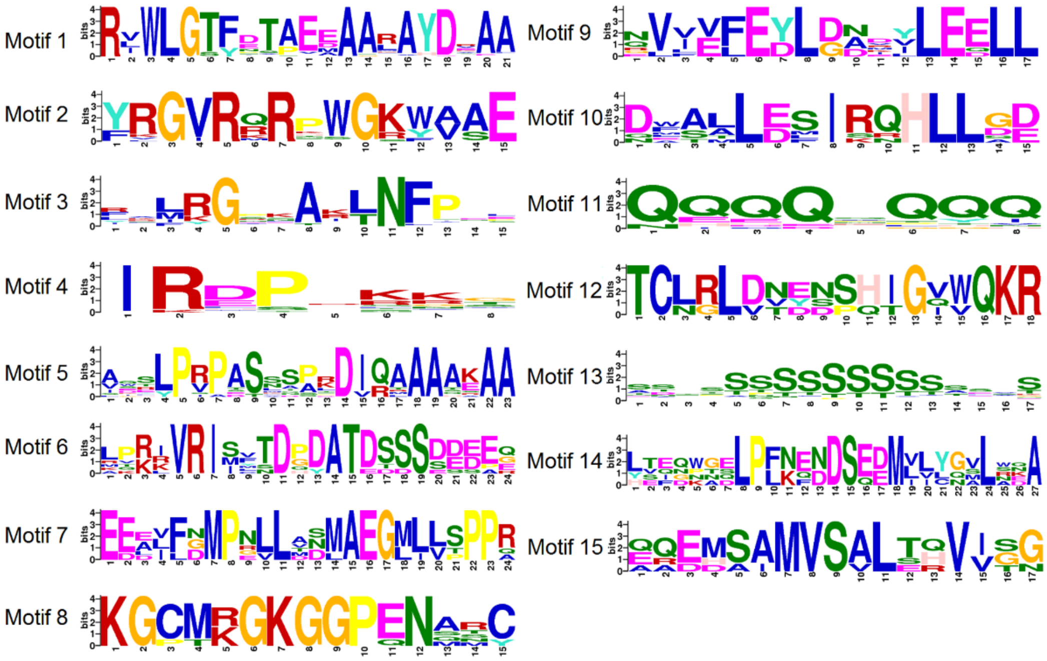

Fig. S4 Sequence logos of FveERF proteins. The overall height of the stack indicates the level of sequence conservation. Height of residues within a stack indicates the relative frequency of each residue at the position.

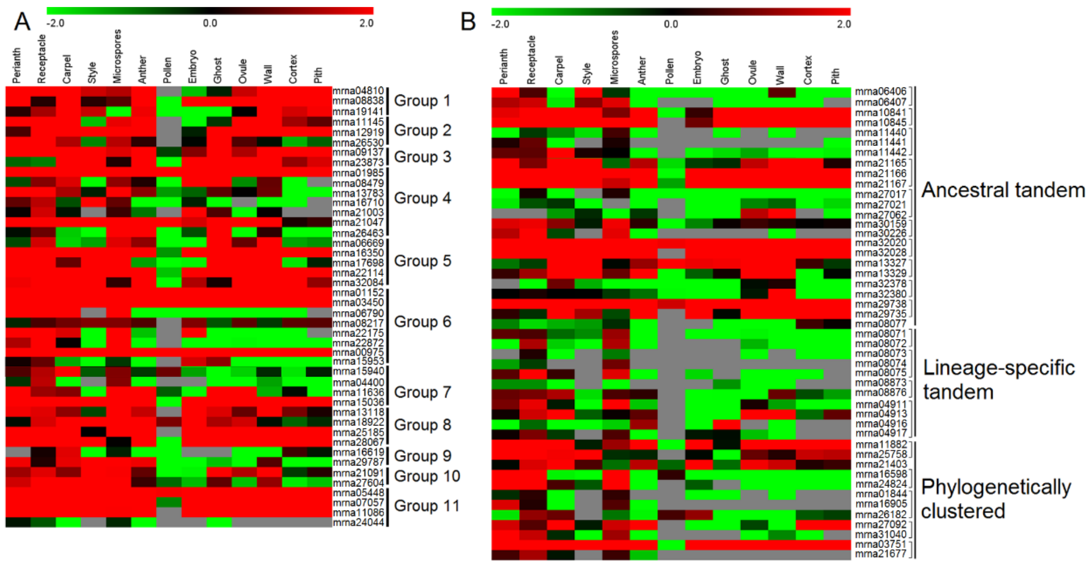

Fig. S5 Expression profiles of *FveERF* genes in different tissues of *F. vesca* flowers and early-stage fruits. **a** and **b** The mRNA levels of the non-tandem (**a**) and tandem/phylogenetically-clustered (**b**) *FveERF* genes. Genes located in a same tandem repeat or in a phylogenetic cluster are grouped together. Mrna08077 forms an ancestral tandem repeat with mrna08071 - mrna08075. Data were retrieved from http://bioinformatics.towson.edu/strawberry/ (Darwish et al. 2013; Kang et al. 2013; Hollender et al. 2014). Expression levels were calculated in the log2 scale. For detailed description of the stages, please see http://bioinformatics.towson.edu/strawberry/newpage/Tissue_Description.aspx.

Table S1 Primers used in this study

Name	Oligonucleotides sequences
GAPDH2 - Forward primer	CATTCATCACCACCGACTACA
GAPDH2 - Reverse primer	GAAGGGTCTTCTCATCCTTGAC
mrna04911 - Forward primer	TCAAACTCGAACTGGGCTATG
mrna04911 - Reverse primer	CGAGTCGTTGCTCGATACTT
mrna04913 - Forward primer	TAAGAGGATGAGGAGGAGTGG
mrna04913 - Reverse primer	ATTCGTCGCCAAATGTGAATG
mrna04916 - Forward primer	CAGTGGATGACGTCGAGATTAAA
mrna04916 - Reverse primer	GTTCTTCGCCGGATCTCTTATC
mrna04917 - Forward primer	GGATCGCTTCCGGTTAGAATAA
mrna04917 - Reverse primer	CAGCTCTCCGAAGTTGAAGAC
mrna08873 - Forward primer	CCTCTTCTCCAAAGACATGGAA
mrna08873 - Reverse primer	GTTGGTTTCTGCTCACTACCT
mrna08876 - Forward primer	GAAATCAGGGAGCCGAGAAA
mrna08876 - Reverse primer	TGCTAGCTCAGGGAAGTTTAAG
mrna06406 - Forward primer	TCTTCCATGCCATCTCATCATC
mrna06406 - Reverse primer	CAGAGGAGTCTCTCCTGTACTT
mrna06407 - Forward primer	CCAGCCTGCTACATCTTCATATC
mrna06407 - Reverse primer	TGAACCGAAATCCGGCATAG
mrna08071 - Forward primer	GTTTGAGGTACATCTTCCTGGTC
mrna08071 - Reverse primer	GCGAACGAAGGTTTGGTAATTG
mrna08072 - Forward primer	AGCATGGTTTGCCAGATAGT
mrna08072 - Reverse primer	CTTCTGGGTTCTGTCCTTGTAG
mrna08075 - Forward primer	GCTCTTTCGTTGCATCACATTC
mrna08075 - Reverse primer	ATCAATCGACCTTCCTCGTTTC
mrna11440 - Forward primer	GACTTTCAACACTGCGGAAGAA
mrna11440 - Reverse primer	GTCCCGGAAGGATACTCGTTAG
mrna11441 - Forward primer	CTCCTCCTTATCCTCCAAGATTTC
mrna11441 - Reverse primer	CAGTTGAGCTACTTCCACTCTC
mrna11442 - Forward primer	CGTGACATGAACTGTGCTACT
mrna11442 - Reverse primer	ACCTCACATCTCTCTCCATCTT
mrna10841 - Forward primer	CCGTGCTTGACGGAGAATTG
mrna10841 - Reverse primer	CGTCTCTTAGAACGCCGTAGA
mrna10845 - Forward primer	CCTCTAGAAGCCGGGAAATATG
mrna10845 - Reverse primer	CTCCTCCGATACCGTTACTTTC
mrna21166 - Forward primer	GAGCTTCATCTCTAGCCTCAAC
mrna21166 - Reverse primer	GTTCAGAAGGGAGCAAGTAGTC
mrna21167 - Forward primer	GAGACTTCACCTTCACAGACAA
mrna21167 - Reverse primer	TTGATGATGGGTTGGAAGTAGAG

Table S2 Classification of the AP2/ERF superfamily in *F. vesca* and *A. thaliana*

Family	*F. vesca*		*A. thaliana*	
	Group	Gene number	Group[a]	Gene number
ERF	1	15	III	22
	2	4	IIb + IIc	9
	3	2	IIa	6
	4	7	IV	9
	5	5	I	10
	6	11	VIII	15
	7	11	V	5
	8	8	VI	12
	9	20	IX	17
	10	4	Xa + Xc	7
	11	4	VII + Xb	9
AP2	-	18	-	18
RAV	-	5	-	6
Soloist	-	1	-	1
Total	-	115	-	146

[a] Grouping of the *Arabidopsis* ERF family is according to Nakano et al. (2006).

Table S3 Information of *FveERF* genes in *F. vesca*

Name	Type	Tandem or Phylogenetic cluster	Group	Chromosome	Number of introns	Duplication mechanism
mrna27017	II	Tandem 1	1	LG5	2	Ancestral tandem
mrna27021[a]	II	Tandem 1	1	LG5	3	Ancestral tandem
mrna27062[a]	II	Tandem 1	2	LG5	0	Ancestral tandem
mrna30159	II	Tandem 2	1	LG6	3	Ancestral tandem
mrna30226	II	Tandem 2	1	LG6	0	Ancestral tandem
mrna32378[a]	II	Tandem 3	1	LG5	0	Ancestral tandem
mrna32380[a]	II	Tandem 3	1	LG5	0	Ancestral tandem
mrna29738[a]	II	Tandem 4	6	LG3	0	Ancestral tandem
mrna29735[a]	I	Tandem 4 &Phylogenetic cluster 10	6	LG3	0	Ancestral tandem
mrna06406	II	Tandem 5	10	LG5	2	Ancestral tandem
mrna06407	II	Tandem 5	10	LG5	2	Ancestral tandem
mrna13327[a]	II	Tandem 6	1	LG7	0	Ancestral tandem
mrna13329[a]	II	Tandem 6	1	LG7	0	Ancestral tandem
mrna32020	II	Tandem 7	8	LG5	8	Ancestral tandem
mrna32028	II	Tandem 7	8	LG5	0	Ancestral tandem
mrna10841[a]	II	Tandem 8	9	LG5	0	Ancestral tandem
mrna10845[a]	II	Tandem 8	9	LG5	0	Ancestral tandem
mrna11440[a]	II	Tandem 9	9	LG4	0	Ancestral tandem
mrna11441[a]	II	Tandem 9	9	LG4	0	Ancestral tandem
mrna11442[a]	II	Tandem 9	9	LG4	1	Ancestral tandem
mrna21165	II	Tandem 10	9	LG7	0	Ancestral tandem
mrna21166[a]	II	Tandem 10	9	LG7	0	Ancestral tandem
mrna21167[a]	II	Tandem 10	9	LG7	0	Ancestral tandem
mrna08077[a]	II	Tandem 11	9	LG2	0	Ancestral tandem

mrna08071[a]	I	Tandem 11 & Phylogenetic cluster 1	9	LG2	1	Ancestral & lineage-specific tandem
mrna08072[a]	I	Tandem 11 & Phylogenetic cluster 1	9	LG2	1	Ancestral & lineage-specific tandem
mrna08073[a]	I	Tandem 11 & Phylogenetic cluster 2	9	LG2	0	Ancestral & lineage-specific tandem
mrna08074[a]	I	Tandem 11 & Phylogenetic cluster 2	9	LG2	0	Ancestral & lineage-specific tandem
mrna08075[a]	I	Tandem 11 & Phylogenetic cluster 2	9	LG2	0	Ancestral & lineage-specific tandem
mrna04911	I	Tandem 13 & Phylogenetic cluster 3	9	LG7	0	Lineage-specific tandem
mrna04913	I	Tandem 13 & Phylogenetic cluster 3	9	LG7	0	Lineage-specific tandem
mrna04916	I	Tandem 13 & Phylogenetic cluster 3	9	LG7	0	Lineage-specific tandem
mrna04917	I	Tandem 13 & Phylogenetic cluster 3	9	LG7	0	Lineage-specific tandem
mrna08873	I	Tandem 14 & Phylogenetic cluster 4	1	LG5	0	Lineage-specific tandem
mrna08876	I	Tandem 12 & Phylogenetic cluster 4	1	LG5	0	Lineage-specific tandem
mrna11882	I	Phylogenetic cluster 5	1	LG5	0	Segmental
mrna25758	I	Phylogenetic cluster 5	1	LG6	0	Segmental
mrna16598	I	Phylogenetic cluster 6	7	LG6	0	Dispersed
mrna24824	I	Phylogenetic cluster 6	7	LG3	0	Segmental
mrna01844	I	Phylogenetic cluster 7	7	LG5	0	Dispersed
mrna16905	I	Phylogenetic cluster 7	7	LG4	0	Dispersed
mrna26182	I	Phylogenetic cluster 7	7	LG5	0	Dispersed
mrna27092	I	Phylogenetic cluster 8	7	LG5	1	Dispersed
mrna31040	I	Phylogenetic cluster 8	7	LG1	1	Segmental
mrna03751	I	Phylogenetic cluster 9	8	LG4	0	Segmental
mrna21677	I	Phylogenetic cluster 9	8	LG4	0	Dispersed
mrna21403	I	Phylogenetic cluster 10	6	LG7	0	Dispersed
mrna04810	II	/	1	LG7	0	ND[b]
mrna08838	II	/	1	LG2	0	ND
mrna19141	II	/	1	LG7	0	ND

mrna11145	II	/	2	LG2	0	ND
mrna12919	II	/	2	LG1	0	ND
mrna26530	II	/	2	LG1	0	ND
mrna09137	II	/	3	LG2	0	ND
mrna23873	II	/	3	LG6	0	ND
mrna01895	II	/	4	LG6	0	ND
mrna08479	II	/	4	LG2	0	ND
mrna13783	II	/	4	LG6	1	ND
mrna16710	II	/	4	LG6	0	ND
mrna21003	II	/	4	LG7	0	ND
mrna21047	II	/	4	LG7	0	ND
mrna26463	II	/	4	LG7	0	ND
mrna06669	II	/	5	LG4	0	ND
mrna16350	II	/	5	LG1	0	ND
mrna17698	II	/	5	LG6	0	ND
mrna22114	II	/	5	LG5	0	ND
mrna32084	II	/	5	LG5	0	ND
mrna00975	II	/	6	Unanchored	0	ND
mrna01152	II	/	6	LG6	0	ND
mrna15953	II	/	6	LG6	1	ND
mrna03450	II	/	6	LG3	3	ND
mrna06790	II	/	6	LG4	0	ND
mrna08217	II	/	6	LG2	1	ND
mrna22175	II	/	6	LG4	0	ND
mrna22872	II	/	6	LG4	0	ND
mrna04400	II	/	7	LG6	0	ND

mrna11636	II	/	7	Unanchored	1	ND
mrna15036	II	/	7	LG2	1	ND
mrna15940	II	/	7	LG6	2	ND
mrna13118	II	/	8	LG7	0	ND
mrna18922	II	/	8	LG7	0	ND
mrna25185	II	/	8	LG5	0	ND
mrna28067	II	/	8	LG3	0	ND
mrna16619	II	/	9	LG6	0	ND
mrna29787	II	/	9	LG3	2	ND
mrna21091	II	/	10	LG1	1	ND
mrna27604	II	/	10	LG2	1	ND
mrna05448	II	/	11	LG6	1	ND
mrna07057	II	/	11	LG4	2	ND
mrna11086	II	/	11	LG2	2	ND
mrna24044	II	/	11	LG6	0	ND

[a] Tandem *FveERF* genes that have tandem *AtERF* orthologs

[b] ND indicates not identified.

Table S4 Correlation coefficients between expression levels of tandem or phylogenetically-clustered *FveERF* duplicates in flowers and early fruits

Duplication type	Gene pair	Correlation coefficient	
		Flower	Early fruit
Ancestral tandem	mrna06406&mrna06407	0.418	-[a]
	mrna10841&mrna10845	0.089	0.612
	mrna11440&mrna11441	-0.114	-
	mrna11440&mrna11442	-0.624	-
	mrna11441&mrna11442	0.847	-
	mrna13327&mrna13329	0.393	0.918
	mrna21165&mrna21166	0.652	0.765
	mrna21165&mrna21167	0.627	0.780
	mrna21166&mrna21167	0.852	0.971
	mrna27017&mrna27021	0.440	-1
	mrna27017&mrna27062	-0.992	-1
	mrna27021&mrna27062	-0.548	1
	mrna30159&mrna30226	0.858	-
	mrna32020&mrna32028	0.821	-0.298
	mrna32378&mrna32380	0.794	1
	mrna29738&mrna29735	0.300	-0.268
	mrna08071&mrna08073	-0.800	-
	mrna08071&mrna08074	-0.968	-
	mrna08071&mrna08075	-0.869	-
	mrna08071&mrna08077	0.506	-
	mrna08072&mrna08073	0.999	-
	mrna08072&mrna08074	0.927	-
	mrna08072&mrna08075	0.993	-
	mrna08072&mrna08077	-0.918	-
	mrna08073&mrna08077	-0.922	-
	mrna08074&mrna08077	-0.704	-
	mrna08075&mrna08077	-0.866	-
Lineage-specific tandem	mrna04911&mrna04913	-0.538	-
	mrna04911&mrna04916	-0.616	-
	mrna04911&mrna04917	0.868	-
	mrna04913&mrna04916	0.995	-
	mrna04913&mrna04917	-0.826	-
	mrna04917&mrna04916	-0.863	-
	mrna08873&mrna08876	0.974	-
	mrna08071&mrna08072	-0.806	-
	mrna08073&mrna08074	0.923	-
	mrna08073&mrna08075	0.991	-
	mrna08074&mrna08075	0.964	-
Phylomrnatically-clustered	mrna11882& mrna25758	0.834	0.79
	mrna21403& mrna29735	0.796	0.944

	mrna27092& mrna31040	-0.257	-
	mrna16598&mrna24824	0.829	-
	mrna01844&mrna16905	-0.368	-
	mrna01844& mrna26182	0.998	-
	mrna16905& mrna26182	-0.414	-
	mrna03751&mrna21677	-0.889	-

[a] Either or both genes have a RPKM value lower than 0.3, thus were regarded not to be expressed and were excluded from the comparison.

Table S5 Expression levels of nine ancestral and nine lineage-specific tandem *FveERF* genes under cold or drought stress

Gene	Drought				Cold			
	0h	1h	3h	8h	0h	1h	3h	8h
mrna06406	0.0112	0.0026	0.0020	0.0030	0.0043	0.0040	0.0047	0.0034
mrna06407	0.0006	0.0011	0.0011	0.0011	0.0009	0.0008	0.0010	0.0007
mrna10841	0.0467	0.1056	0.0621	0.0574	0.0753	0.0667	0.0685	0.0253
mrna10845	0.0164	0.1323	0.0666	0.0684	0.0188	0.0470	0.0807	0.0260
mrna11440	9.91E-05	6.21E-05	5.60E-05	8.01E-05	0.0004	0.0002	0.0001	0.0004
mrna11441	0.0014	0.0005	0.0005	0.0006	0.0010	0.0010	0.0016	0.0016
mrna11442	0.0040	0.0042	0.0045	0.0034	0.0065	0.0055	0.0060	0.0031
mrna21166	0.2474	0.4390	0.2464	0.2559	0.0599	0.1496	0.2144	0.0957
mrna21167	0.1444	0.0910	0.0418	0.0362	0.0301	0.0467	0.0577	0.0369
mrna04911	1.79E-06	6.36E-06	7.46E-06	7.98E-06	8.68E-07	1.24E-06	1.26E-06	5.82E-06
mrna04913	0.0001	0.0003	0.0004	0.0003	7.26E-05	7.23E-05	9.92E-05	0.0002
mrna04916	0.0031	0.0005	0.0006	0.0017	0.0009	0.0005	0.0006	0.0003
mrna04917	0.0008	0.0001	0.0002	0.0009	0.0006	0.0003	0.0002	0.0002
mrna08873	0.0003	0.0002	0.0001	0.0002	0.0001	0.0001	0.0001	0.0003
mrna08876	8.32E-06	1.14E-05	7.55E-06	4.40E-06	0.0003	0.0002	0.0001	0.0003
mrna08071	1.09E-05	0.0009	0.0002	0.0002	1.08E-05	6.40E-05	7.74E-05	3.16E-05
mrna08072	0.0001	0.0008	0.0002	0.0001	3.18E-05	1.73E-05	2.20E-05	1.39E-05
mrna08075	0.0002	0.0002	0.0002	0.0001	2.55E-05	4.73E-05	0.0001	5.75E-05

The expression levels were measured by quantitative RT-PCR using *GAPDH* as the reference gene. Three biological replicates and three technical replicates were obtained for each data point.

Table S6 Correlation coefficients between expression levels of tandem *FveERF* duplicates under stress conditions

Duplication type	Gene pair	Correlation coefficient	
		Drought	Cold
Ancestral tandem	gene06407&gene06407	0.469	0.962
	gene10841&gene10845	0.954	0.301
	gene11440&gene11441	-[a]	-0.087
	gene11440&gene11442	-	-0.602
	gene11441&gene11442	-0.193	-0.620
	gene21166&gene21167	0.147	1
	gene08071&gene08075	0.248	-
	gene08072&gene08075	0.358	-
Lineage-specific tandem	gene04913&gene04916	-0.975	-
	gene04913&gene04917	-0.620	-
	gene04916&gene04917	0.777	0.911
	gene08873&gene08876	-	0.853
	gene08071&gene08072	0.988	-

[a] Expression levels of either or both genes are too low to be detected, so the gene pair were excluded from the comparison.

Table S7 Pairwise nucleotide divergence, Ka, Ks and Ka/Ks values of tandem *FveERF* duplicates

Duplicates	Gene pair	Pi	Ka	Ks	Ka/Ks
Lineage-specific tandem	mrna08873&mrna08876	0.2553	0.2603	0.6029	0.4317
	mrna04911&mrna04913	0.147	0.1335	0.5248	0.2543
	mrna04911&mrna04916	0.3566	0.4023	1.8412	0.2185
	mrna04911&mrna04917	0.4546	0.3475	2.2054	0.1576
	mrna04913&mrna04916	0.3517	0.3717	1.998	0.186
	mrna04913&mrna04917	0.4401	0.3333	2.0541	0.1623
	mrna08073&mrna08074	0.6265	0.262	0.8043	0.3257
	mrna08073&mrna08075	0.5174	0.2182	3.0074	0.0726
	mrna08074&mrna08075	0.5142	0.2777	1.2891	0.2154
	mrna08071&mrna08072	0.2807	0.347	0.5398	0.6428
	Average	0.3944	0.2954	1.4867	0.2667
Ancestral tandem	mrna29735&mrna29738	0.5809	0.5544	2.8273	0.1961
	mrna10841&mrna10845	0.6259	0.838	3.949	0.2122
	mrna13327&mrna13329	0.5507	0.6422	3.7566	0.171
	mrna21165&mrna21166	0.6385	0.8485	2.2655	0.3745
	mrna21165&mrna21167	0.5981	0.8907	2.2049	0.4039
	mrna21166&mrna21167	0.5818	0.5367	1.5956	0.3364
	mrna30159&mrna30226	0.6304	0.64	3.3327	0.192
	mrna08073&mrna08071	0.4842	0.5392	3.3927	0.1589
	mrna08073&mrna08072	0.4318	0.6131	1.5709	0.3903
	mrna08073&mrna08077	0.5191	0.5856	2.6937	0.2174
	mrna08074&mrna08071	0.5024	0.5869	3.5788	0.1639
	mrna08074&mrna08072	0.4556	0.6131	3.1563	0.1942
	mrna08074&mrna08077	0.5594	0.6382	2.1349	0.2989
	mrna08075&mrna08071	0.5539	0.6056	3.4298	0.1766
	mrna08075&mrna08072	0.4747	0.5903	2.6004	0.227
	mrna08075&mrna08077	0.5608	0.6233	2.702	0.2307
	mrna08071&mrna08077	0.5201	0.73	2.2995	0.3175
	mrna08072&mrna08077	0.529	0.6972	1.7629	0.3955
	mrna11440&mrna11441	0.4127	0.359	3.3994	0.1056
	mrna11440&mrna11442	0.5123	0.5819	2.299	0.2531
	mrna11441&mrna11442	0.5373	0.5768	3.5252	0.1636
	mrna27017&mrna27021	0.5513	0.3636	1.3669	0.266
	mrna27017&mrna27062	0.7126	0.8728	3.0849	0.2829
	mrna27021&mrna27062	0.6128	0.7306	3.4171	0.2138
	mrna32020&mrna32028	0.538	0.3129	0.951	0.329
	mrna06406&mrna06407	0.3465	0.4759	1.1103	0.4286
	Average	0.5392^{***}	0.6176^{***}	2.6311^{***}	0.2578

*** indicates a significance value $p < 0.001$ between the ancestral and lineage-specific tandem groups.

References

Darwish, O., Slovin, J. P., Kang, C., Hollender, C. A., Geretz, A., and Houston, S., et al. (2013). SGR: an online genomic resource for the woodland strawberry. *BMC Plant Biol.* 13:223.

Hollender, C. A., Geretz, A. C., Slovin, J. P., and Liu, Z. (2012). Flower and early fruit development in a diploid strawberry, *Fragaria vesca*. *Planta* 235, 1123–1139. doi: 10.1007/s00425-011-1562-1

Kang, C., Darwish, O., Geretz, A., Shahan, R., Alkharouf, N., and Liu, Z. (2013). Genome-scale transcriptomic insights into early-stage fruit development in woodland strawberry *Fragaria vesca*. *Plant Cell* 25, 1960–1978. doi: 10.1105/tpc.113.111732

Nakano T, Suzuki K, Fujimura T et al (2006). Genome-wide analysis of the ERF gene family in Arabidopsis and rice. *Plant Physiol.* 140, 411–432
